# Assessment of prediction methods for protein structures determined by NMR in CASP14: Impact of AlphaFold2

**DOI:** 10.1002/prot.26246

**Published:** 2021-10-19

**Authors:** Yuanpeng Janet Huang, Ning Zhang, Beate Bersch, Krzysztof Fidelis, Masayori Inouye, Yojiro Ishida, Andriy Kryshtafovych, Naohiro Kobayashi, Yutaka Kuroda, Gaohua Liu, Andy LiWang, G.V.T Swapna, Nan Wu, Toshio Yamazaki, Gaetano T. Montelione

**Affiliations:** ^1^ Department of Chemistry and Chemical Biology Center for Biotechnology and Interdisciplinary Sciences, Rensselaer Polytechnic Institute Troy New York USA; ^2^ Department of Chemistry and Biochemistry University of California Merced California USA; ^3^ Biomolecular NMR Spectroscopy Group Institut de Biologie Structurale, UMD‐5075, CNRS‐CEA‐UJF Grenoble France; ^4^ Genome Center University of California Davis California USA; ^5^ Department of Biochemistry Robert Wood Johnson Medical School, Rutgers University Piscataway New Jersey USA; ^6^ Center for Advanced Biotechnology and Medicine Rutgers University Piscataway New Jersey USA; ^7^ NMR Science and Development Division RSC, RIKEN Yokohama Kanagawa Japan; ^8^ Department of Biotechnology and Life Science Graduate School of Engineering, Tokyo University of Agriculture and Technology (TUAT) Tokyo Japan; ^9^ Nexomics Biosciences, Inc. Rocky Hill New Jersey USA; ^10^ Center for Cellular and Biomolecular Machines and Health Sciences Research Institute University of California Merced California USA; ^11^ Department of Pharmacology Robert Wood Johnson Medical School, Rutgers University Piscataway New Jersey USA; ^12^ College of Food and Bioengineering Zhengzhou University of Light Industry Zhengzhou China

**Keywords:** integral membrane proteins, structure determination, machine leaning, MipA, protein dynamics, protein structure prediction, solution NMR

## Abstract

NMR studies can provide unique information about protein conformations in solution. In CASP14, three reference structures provided by solution NMR methods were available (T1027, T1029, and T1055), as well as a fourth data set of NMR‐derived contacts for an integral membrane protein (T1088). For the three targets with NMR‐based structures, the best prediction results ranged from very good (GDT_TS = 0.90, for T1055) to poor (GDT_TS = 0.47, for T1029). We explored the basis of these results by comparing all CASP14 prediction models against experimental NMR data. For T1027, NMR data reveal extensive internal dynamics, presenting a unique challenge for protein structure prediction methods. The analysis of T1029 motivated exploration of a novel method of “inverse structure determination,” in which an AlphaFold2 model was used to guide NMR data analysis. NMR data provided to CASP predictor groups for target T1088, a 238‐residue integral membrane porin, was also used to assess several NMR‐assisted prediction methods. Most groups involved in this exercise generated similar beta‐barrel models, with good agreement with the experimental data. However, as was also observed in CASP13, some pure prediction groups that did not use any NMR data generated models for T1088 that better fit the NMR data than the models generated using these experimental data. These results demonstrate the remarkable power of modern methods to predict structures of proteins with accuracies rivaling solution NMR structures, and that it is now possible to reliably use prediction models to guide and complement experimental NMR data analysis.

## 1 INTRODUCTION

The remarkable performance of some protein structure prediction groups in the 2020 Critical Assessment of Protein Structure Prediction experiment 14 (CASP14) has set a new standard for protein structure modeling.^1^ These breakthrough technologies exploit advances in attention‐based machine learning,^2^, ^3^ contact prediction based on sequence co‐variance analysis using the massive data bases of genomic sequence data,^4^, ^5^, ^6^, ^7^, ^8^, ^9^ and the rapidly growing database of experimental protein structures. In particular, in blind tests of protein structure prediction accuracy on 96 CASP14 targets, the performance of DeepMind AlphaFold2 (AF2)^10^ had an unprecedented high accuracy, assessed by backbone atomic coordinate global distance test (GDT_TS) scores,^11^ of 0.88 ± 0.1, corresponding to a backbone atom root‐mean‐squared deviation (RMSD) between predicted and experimental protein structures of about 1.5 Å.^1^ Buried sidechain conformations in these blind predictions of protein structure are also generally a remarkable good match between the predicted model and experimental structure.^12^

In the previous 2018 CASP13 experiment, we explored the concept of using incomplete “sparse” solution NMR data to assist protein structure prediction methods.^13^ The aim of this earlier study was to assess if advanced structure prediction methods could be combined with the kinds of sparse NMR data that can be obtained on medium‐sized (20–50 kDa) proteins, which are otherwise challenging for structure determination by solution NMR. NOESY data typical of that easily obtained for ^15^N,^13^C‐enriched, perdeuterated proteins up to about 40 kDa, were simulated for 11 CASP13 targets ranging in size from 80 to 326 residues, and used to generate tables of ambiguous contacts using simple NOESY peak assignment protocols. These ambiguous contact lists were provided, together with simulated ^15^N‐^1^H residual dipolar coupling (RDC) data and backbone dihedral angle restraints obtainable from chemical shift data, to the CASP prediction community for data‐assisted prediction. Real NMR data collected for a de novo designed protein were also used to generate ambiguous contact tables and chemical‐shift based backbone dihedral angle restraints, that were also provided to CASP13 predictor groups, including one set of (ambiguous) NMR‐based contacts in which only backbone resonance (no sidechain) assignments were available. Guided by these “sparse” experimental NMR data, some CASP13 prediction groups generated models more accurate than those produced using more traditional protein NMR modeling methods.^13^

The best NMR‐assisted models were also compared with the best “regular” prediction (i.e., pure prediction) models provided by all CASP13 groups. For 6 of 13 target data sets, the most accurate model provided by any NMR‐assisted prediction group was more accurate than the most accurate model provided by any regular prediction group, as expected. However, for the remaining 7 target data sets, one or more regular prediction method provided a more accurate model than even the best NMR‐assisted model. Here, accuracy was assessed by comparison with the reference X‐ray crystal structure from which ambiguous contacts were derived, or the experimental NMR structure determined with a much larger amount of NMR data. Hence, for some of these blind structure predictions, pure prediction methods provided more accurate models than either traditional NMR structure determination or data‐assisted prediction methods that used these simulated or real sparse NMR data.^13^ Machine learning methods, and particularly the AlphaFold methods (the progenitor of AlphaFold2), were particularly successful in CASP13, providing accurate models even without any experimental data.^13^

In CASP14, three reference structures provided by solution NMR methods were available (targets T1027, T1029, and T1055), as well as a fourth data set of NMR‐derived contacts for NMR data‐assisted structure prediction (T1088). For the three CASP14 targets with reference structures provided by solution NMR methods, the best AF2 prediction results range from very good (GDT_TS_best = 0.90, for T1055), to medium (GDT_TS_best = 0.67, for T1027), to poor (GDT_TS_best = 0.47, for T1029). We explored the basis of these results by comparing ^1^H–^1^H distance maps derived from these models against the experimental NOESY peak lists using recall and precision scores (RPF‐DP scores).^14^, ^15^ Models were also compared with backbone chemical shift data using the *TALOS_N* program,^16^ and RDC data where available. These results demonstrate the remarkable accuracy of some CASP14 prediction models, particularly AlphaFold2, and reveal different reasons for the differences between experimental and prediction models for each target for which the reference struture was determined by NMR methods.

## 2 METHODS

### 2.1 Knowledge‐based structure validation

Structure quality assessment included analysis of knowledge‐based structure quality scores, including Ramachandran backbone analysis,^17^ *ProCheck* dihedral angle analysis for both backbone dihedral angles and all dihedral angles (i.e., backbone and sidechain),^18^ *ProsaII*,^19^ *Verify3D*,^20^ and *Molprobity*,^21^ using the Protein Structure Validation Software suite (PSVS) server.^22^ Knowledge‐based dihedral angle analysis was restricted to well‐defined residues, defined by the method of *Cyrange* ^23^ as recommended by the wwPDB NMR structure validation task force.^24^ For each of these knowledge‐based structure quality assessment metrics, *Z* scores are reported relative to the corresponding raw scores obtained for a set of 252 X‐ray crystal structures each of <500 residues, and with resolution ≤ 1.8 Å, R factor ≤ 0.25, and R‐free ≤ 0.28^22^; positive *Z* scores correspond to knowledge‐based structure quality scores better than the average score in this set of reference structures. Generally speaking, acceptable NMR‐based models have *Z* scores > −3.0 for *ProCheck* (backbone), *ProCheck* (backbone plus sidechain), *ProsaII*, and *MolProbity*,^22^ while *Verify3D* scores for accurate structures are more variable and dependent on the protein fold, but generally have *Z* scores > −5.0.

### 2.2 NMR restraint violation analysis

NMR distance and restraint violations were assessed consistently using experimental distance restraint lists generated by different programs and available in the Protein Data Bank using the *PDBStat* software.^25^ Model agreement with backbone chemical shift data deposited in the BioMagResDatabase was assessed using the *Talos_N* program.^16^

### 2.3 RDC *Q* scores

The RDC *Q* score (or quality factor)^26^ was used to quantify the extent of agreement between a structure and measured dipolar couplings. A *Q* score below 0.2 can be used as a rule of thumb to indicate adequate agreement between the model and the RDC data. *Q* scores are calculated using the following equation:

$$Q\;\text{score}=\sqrt{\frac{\sum_{i}W^{2}{\left(D_{i,\mathrm{obs}}-D_{i,\mathrm{cal}}\right)}^{2}}{N*D_{a}^{2}\left(4+3R_{h}^{2}\right)/5}}$$

*W* is the weighting factor; *N* is the number of dipolar couplings; *D*_a_ is the axial component of the orientation tensor; and *R*_*h*_ is the rhombicity of the orientation tensor. RDCs and *Q* scores were back‐calculated from models using single‐value decomposition tools available on DC: Servers for Dipolar Coupling Calculations (https://spin.niddk.nih.gov/bax/nmrserver/dc/svd.html).

### 2.4 RPF‐DP scores for CASP14 NMR structures and prediction models

RPF‐DP scores are a set of fast and sensitive structure quality assessment measures which can be used to evaluate how well a 3D structure model fits with NOESY peak and chemical shift data, to assess the correctness of the fold and accuracy of the structure.^14^, ^15^ RPF‐DP scores provide a type of NMR R‐factor, in which models are compared against NMR NOESY data. They have been described previously,^14^, ^15^ but as they play a key role in this work, we provide an overview of these model versus data structure quality assessment metrics here.

**FIGURE 1:**
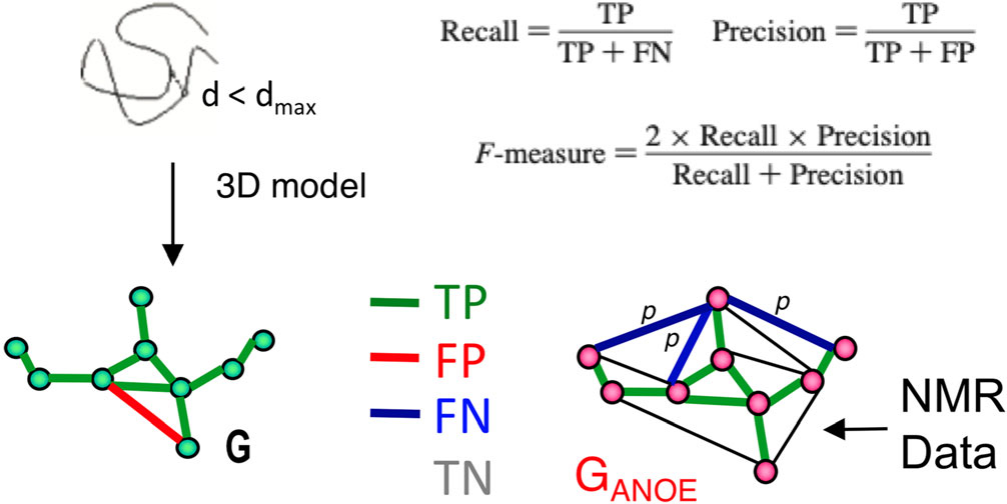
DP scores in CASP14. Schematic description of RPF‐DP scores. In this analysis, the graph G with nodes corresponding to all assigned ^1^H's and edges representing all short (<5 Å) ^1^H–^1^H distances in a structure model (left), is compared with a graph G_ANOE_ (right), in which nodes again correspond to all assigned ^1^H's and edges describe all possible assignments for each NOESY cross peak. TPs are edges common to both G and G_ANOE_, false positives (FPs) are edges present in G but not in G_ANOE_, and false negatives (FNs) are the set of edges in G_ANOE_ representing the multiple possible assignments of a NOESY cross peak, none of which are present in G. These metrics are used to compute recall (R), precision (P), and F‐measure as shown in the figure and outlined in the Methods Section. The F‐measure is the harmonic mean of the recall and precision. The Discriminating Power (DP) is a normalized F‐measure corrected to account for the F‐measure expected for a random‐coil chain (DP = 0) and the best F‐measure possible considering the completeness of the NMR data (DP = 1.0).^15^ Accurate structures generally have DP for individual models > 0.60

The RPF‐DP score algorithm is outlined schematically in Figure 1. Nodes represent all protons listed in the resonance assignment table. Edges connect the nodes and represent all potential associated NOEs from the NOESY peak lists, within a chemical shift match tolerance. In constructing the ambiguous graph G_ANOE_ (shown on right side of Figure 1) each NOESY cross peak (p) may be ambiguously assigned to one or more proton pairs, as determined by chemical shift degeneracies and match tolerances. The solution graph, G_NOE_, corresponding to the true 3D structure, is a subgraph of G_ANOE_. Given complete NOESY peak lists and resonance assignments, for each NOESY cross peak p, at least one of its possible proton pair assignments has a corresponding edge in G_NOE_. For each structure model (shown on left side of Figure 1), a distance network graph G is calculated from the summation distances (sum of inverse sixth powers of individual degenerate proton–proton distances), assuming uniform effects of nuclear relaxation processes. Nodes are connected by an edge in G if the corresponding interproton summation distance in the model structure is ≤ d_NOE_max_, where d_NOE_max_ is the (estimated) maximum distance detected in the NOESY spectrum. Summation distances are used to address the lack of stereospecific assignments of prochiral methylene proton pairs, sets of protons that are degenerate (e.g., the three hydrogens of a methyl group, degenerate methylene protons, or degenerate resonances of Tyr or Phe), or combinations of these kinds of ambiguities (e.g., for prochiral isopropyl methyl groups of Leu or Val for which stereospecific assignments are not available).

NOESY cross peaks represented in G_ANOE_ that are consistent with the short interproton distances in the network derived from the model, G, are defined as true positives (TPs), while NOESY peaks expected from the model (edges in G) that are not observed in the data, G_ANOE_, are true negatives (TNs). As illustrated in Figure 1, particular proton pair interactions present in the atomic coordinates of a model structure, represented by the network G, may either be represented in the graphical representation of the NOESY peak list data G_ANOE_ (TP), or not (FP). Since G_ANOE_ is an ambiguous network, a FN score is assigned to the peak only if none of the several possible short proton–proton distance consistent with all possible NOESY peak assignments are observed in G. In this context, recall measures the fraction of NOE cross peaks that are consistent with the query model structures, while precision measures the fraction of proton pair interactions in the query structure that are observed in the NOESY peak list (i.e., in G_ANOE_), weighted by interproton distance. The F‐measure is the harmonic mean of the recall and precision. Equations used to calculate recall (R), precision (P), and F‐measure (F, also called the performance) are shown in Figure 1.

The DP score is a normalized F‐measure that accounts for lower‐bound and upper‐bound values of the F‐measure. The lower‐bound of F(G) is estimated by F(G_free_), where G_free_ is a distance network graph computed from interproton distances in a freely rotating polypeptide chain model, as described by Flory and co‐workers.^27^ The upper‐bound of F(G) is estimated by F(G_ideal_). G_ideal_ is the graph of a hypothetical ideal structure that is perfectly consistent with G_ANOE_. It is defined so that recall (G_ideal_) = 1 and precision(G_ideal_) = precision(G_local_), where G_local_ is the network of all two and three‐bond connected proton pairs; that is, the completeness of the network G_ANOE_ is assumed to be approximately the same as the completeness of the subnetwork of NOEs associated with these local ^1^H–^1^H distances, G_local_. With these definitions, F(G_ideal_) represents the best possible performance F considering the quality of the input NOESY peak lists and resonance assignments. F(G_ideal_), and particularly the precision of G_ideal_, thus provides a measure of the combined quality of the resonance assignment and NOESY peak lists for one or more spectra. F(G_ideal_) and F(G_free_) describe the two bounds of the performance F(G); that is, F(G_ideal_) ≤ F(G) ≤ F(G_free_). With these definitions, the fold Discriminating Power (DP) for G is then estimated by scaling the F values so that F(G_ideal_) = DP(G_ideal_) = 1, and F(G_free_) = DP(G_free_) = 0. This scaling is necessary to account for the fact that the NOESY data may not be complete, and the observation that even a random coil chain model can satisfy a large part of the NOESY peak list data.^15^

The default upper‐bound observed distance, d_NOE_max_, used in these metrics is 5 Å, but can also be calibrated from the NOESY data. In this analysis, a distance (d^−6^) weighting of the precision metric, precision_w_(G), is used to reduce the otherwise dominant influence of the many weak NOEs arising from interproton distances close to the upper‐bound detection limit, d_NOE_max_. This weighting also makes these quality scores less sensitive to the value chosen for d_NOE_max._ ^15^

RPF‐DP scores can be calculated for individual models, or using average distances across an ensemble. The ensemble DP score is usually 10–15% higher than individual DP scores. In various studies,^14^, ^15^, ^28^, ^29^ structures within 2.0 Å RMSD of the corresponding “correct” structure have been observed to have DP scores > 0.70 for NMR ensembles, and DP scores > 0.60 for individual conformers. Perdeuterated protein NMR data, like that obtained here for MipA, require a larger d_max_ (7 Å), and generally provide somewhat lower RPF‐DP scores.

### 2.5 ANSURR scores

The Accuracy of NMR Structures Using RCI and Rigidity (ANSURR) method provides an independent assessment of model quality by comparing protein flexibility computed from backbone chemical shifts with protein flexibility predicted with a graph theory based measure of structural rigidity.^30^ ANSURR provides two measures of similarity between these measures, a correlation score (corr) which assesses the correlation between peaks and troughs of observed and predicted structural flexibility along the sequence, and root‐mean‐squared deviation (RMSD) between the metrics. Both the corr and RMSD score are reported as a percentile score (ranging from 0 to 100). These scores were calculated using the ANSURR program version 1.0.2 (https://zenodo.org/badge/latestdoi/234519929).

### 2.6 Experimental NOESY peak lists and model preparation

For targets T1027, T1029, and T1055, NOESY peak lists were obtained from the experimentalists who carried out the original NMR structure analyses. For target T1088, experimental data was collected and NOESY peak lists were generated as outlined in Supporting Information Methods. For assessing experimental NMR structures, the coordinates with hydrogen atoms for each conformer are used. For assessing prediction models or X‐ray crystal structures, which generally do not include hydrogen atoms, the program *Reduce* ^31^ was used to add protons with ideal covalent geometries. DP scores were calculated by comparing individual conformers against the NOESY peak and chemical shift lists. We noticed that the program *Reduce* failed to add all protons for some of the CASP14 prediction models, due to their unrealistic heavy atom geometry. The DP scores for these models with physically unreasonable geometry tend to have very small or negative values.

### 2.7 Global distance test scores

GDT_TS scores were computed by the CASP Prediction Center using the method of Zemla.^11^ For brevity, GDT_TS scores are referred to throughout this paper as GDT scores.

### 2.8 Molecular modeling

Molecular modeling was done using *PyMol*.^32^

### 2.9 NMR data for integral membrane protein target MipA in detergent micelles

MipA is an antibiotic‐resistance factor, which acts to transport some drugs out of bacteria, while enhancing transport of other drugs into bacteria.^33^ The expression, isotope‐enrichment, and purification of MipA is outlined in the Supporting Information Methods. Briefly, a synthetic codon‐optimized gene (Genscript, Inc) for *Klebsiella pneumoniae* MipA was expressed using the pColdII single protein expression system.^34^, ^35^ The resulting protein construct includes a short N‐terminal 6xHis purification tag. MipA samples for NMR studies were prepared with ^2^H, ^13^C, ^15^N, and ^13^CH_3_ methyl‐enrichment. MipA was expressed in *Escherichia coli* BL21(DE3)*ΔhisB* cells harboring *pACYCmazF(ΔH)* and *pCold2‐mipA*, solubilized with 8 M urea, purified by Ni‐NTA affinity chromatography, and then refolded by slow removal of urea by dialysis. The purified protein was prepared in 20 mM potassium phosphate buffer at pH 6.5, containing 0.2 M NaCl, 50 mM M Arg, and 0.1% d_37_‐DPC. The resulting sample was >95% homogenous on SDS‐PAGE gels. The final protein concentration for NMR studies was ~0.5 mM. ^2^H‐decoupled NMR studies were carried out using Avance 600 and 800 NMR spectrometer systems located at Rutgers University, Princeton University, and Rensselaer Polytechnic Institute. Details of NMR data collection and processing are provided in Supporting Information Methods. NMR data for MipA were provided to CASP14 predictor groups in the form of Ambiguous Contact Lists, prepared as described previously.^13^ Briefly, for each NOESY cross peak we provide a list of possible assignments by analyzing NOESY peak lists together with the corresponding resonance assignment lists using the Cycle 0 module of the program *ASDP*,^36^ providing a simple matching between resonance frequencies of the NOESY peak and the resonance assignment list. Details of this process are outlined in Supporting Information Methods.

## 3 RESULTS

### 3.1 Target T1055: A20_304_ _‐426_

The A20 protein of vaccinia virus forms a heterodimer processivity factor with the uracil‐DNA glycolase, D4 protein, and binds the catalytic subunit of the DNA polymerase, E9 protein, to form the essential DNA polymerase holoenzyme E9‐A20‐D4 required for viral DNA synthesis. CASP14 target T1055 is the C‐terminal domain of A20, corresponding to the last 123 residues. The construct used for structural studies included a C‐terminal biotin acceptor protein (BPAP) tag, connected by a 10‐residue linker.^37^ The solution NMR structure of A20_304‐426_ was determined by Bersch et al.^38^ from triple‐resonance NMR and NOESY data (*τ*_m_ = 100 ms), which provided 2351 unambiguous and 566 ambiguous distance restraints, together with 218 backbone dihedral angle restraints based on analysis of chemical shift data with *Talos+*. The *UNIO10* program suite^39^ was used for initial NOE assignment. The resulting NOESY peak lists generated with the *ATNOS* peak picking algorithm were then used for structure calculation using *ARIA 2.3*,^40^ followed by refinement with *CNS 1.21*.^41^ Although potential NOEs were observed between the C‐terminal linker‐BPAP purification tag and the core of the structure, these NOEs were excluded from the analysis because of their ambiguity in assignment.^38^ The resulting well‐defined structure (PDB ID 6zyc), reported as an ensemble of 20 conformers, includes 5 N‐terminal α‐helices, a two‐stranded antiparallel β‐sheet, and a long C‐terminal helix. ^15^N relaxation data indicate that A20_304‐426_‐BAP has dynamic flexibility in its N‐terminal ~10‐residue polypeptide segment, and in the C‐terminal linker‐BAP tag, but otherwise has a relatively static overall backbone structure.

**FIGURE 2:**
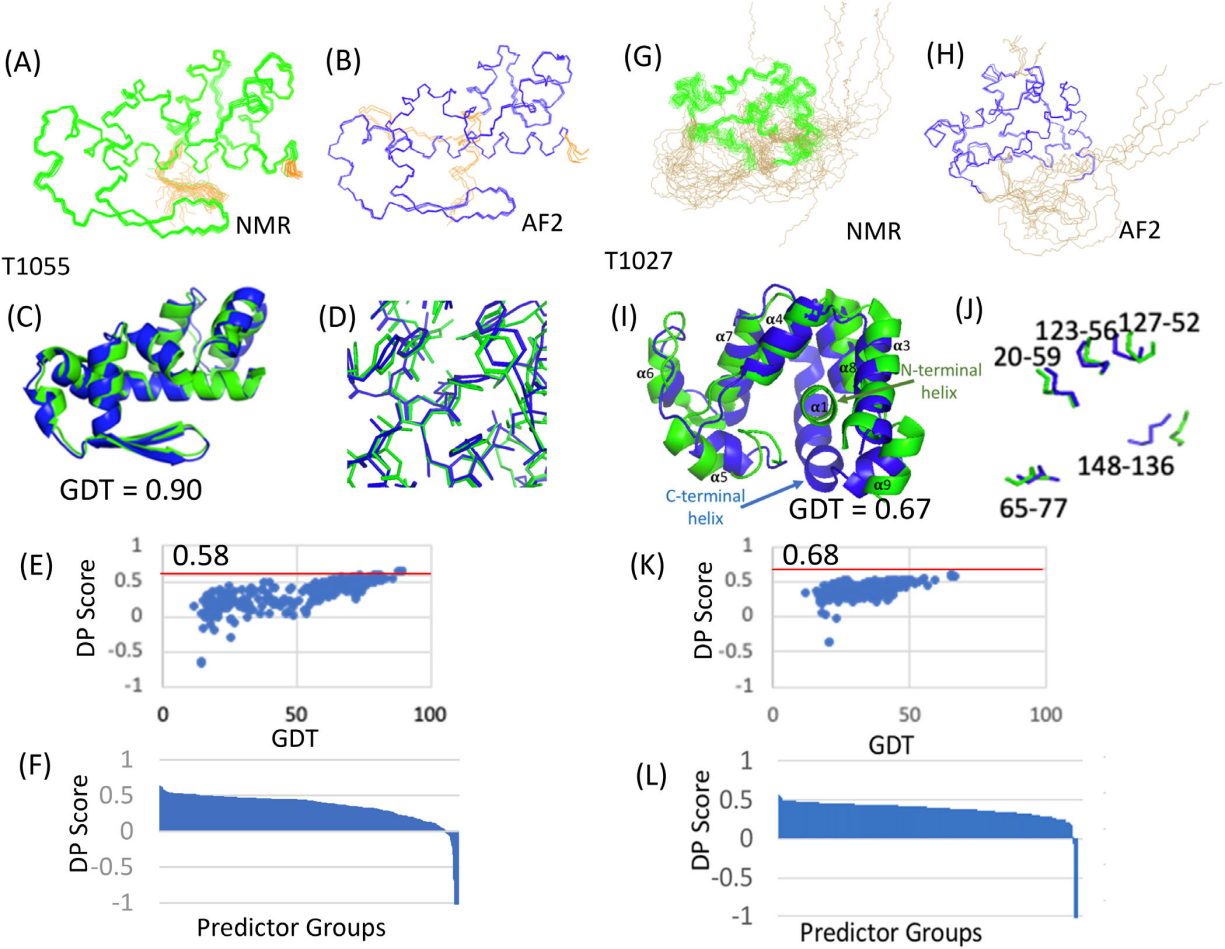
Structural analysis for CASP14 targets 1055 and 1027. (left) Superimposed ensembles for (A) NMR structure (PDB ID 6zyc) (green) and (B) AF2 structures (blue) of T1055, illustrating the not‐well‐defined segments (brown) as defined by *Cyrange*.^23^ For the NMR structure, residues 305–426 are well‐defined, while for the AF2 structure residues 310–428 are well‐defined (residues 427 and 428 being part of the linker to the purification tag). (C,D) Comparison of AF2 conformer with highest GDT score (blue) with the representative conformer from the NMR structure ensemble with best DP score, for residues 310–426 of T1055. The well‐defined backbone (N, C^α^, C′) atoms are superimposed and both the backbone superimposition and associated core sidechains are illustrated. DP versus GDT scores (E) and DP scores versus predictor group (F) for target T1055. (right) Superimposed ensembles for (G) NMR structure (PDB ID 7d2o) (green) and (H) AF2 structure (blue) of T1027, illustrating the not‐well‐defined segments (brown). For the NMR structure, residues 10–18, 36–81, and 96–145 are well‐defined,^45^ while for the AF2 structure residues 36–75 and 96–164 are well‐defined. (I,J) Comparison of AF2 conformer with highest GDT score (blue) with the conformer from the NMR structure ensemble with highest DP score, for T1027. The well‐defined backbone (N, C^α^, C′) atoms are superimposed for residue ranges 36–75 and 96–145. In the NMR structure, the N‐terminal helix (α1) sits in a pocket in the core of the protein, while the C‐terminal region is disordered (and therefore not shown in panel I); while in the AF2 structure, the N‐terminal region is disordered (and not shown in panel I), and the C‐terminal region forms a C‐terminal helix that packs into the core of the protein structure. The five disulfide bonds of T1027 are illustrated in panel J. DP versus GDT scores (K) and DP scores versus predictor group (L). The red horizontal lines in (E) and (K) are drawn at the DP scores of the best scoring conformation from the ensemble of experimental structures. For both targets, only residues that are well‐defined in both the NMR or AF2 structures were included in superimposition and GDT score calculations. The nine helices of the NMR model, as well as the C‐terminal helix of the AF2 model, are labeled in panel I

We assessed the similarities between NMR and CASP14 prediction models, including AF2 models (Figure 2, left). In comparing the predicted AF2 structure of T1055 with the experimental structure, we excluded residue segments for which atomic positions are not well defined in either the ensemble of 20 NMR‐derived conformers or the ensemble of 5 AF2 conformers. Well‐defined regions of the NMR ensemble, residues 305–426 (Figure 2A) and AF2 ensemble, residues 310–426 (Figure 2B), were identified using the program *Cyrange*. Residues 303–313 also have hetNOE values < 0.5. For the residues whose backbone positions are well defined in both ensembles, that is, for residues 310–426, the pairwise GDT scores between the NMR model with best DP score and the 5 AF2 conformers ranged from 0.89 to 0.90, corresponding to backbone RMSD's of about 1.3 Å (Figure 2C). Many buried sidechain conformations also have relatively good agreement between the AF2 and NMR structures (Figure 2D). This is a remarkable result considering that the AF2 prediction did not use any NMR data.

Structure quality statistics for T1055 were also analyzed with the *PSVS* software suite. The resulting *PSVS* structure quality statistics for both the NMR and AF2 model ensembles are summarized in Tables S1 and S2. Both the NMR and AF2 models generally exhibit excellent structure quality scores and good energetics. However, the AF2 models have significantly better *ProCheck* (backbone and sidechain) G‐factor and *Molprobity* clash scores, attributable to more energetically consistent core sidechain packing.

We next assessed how well the NMR and AF2 structures fit to the experimental NMR chemical shift (bmrb_id 34 545) and NOESY peak list data using the RPF‐DP score.^14^, ^15^ Plots of DP score versus GDT for all CASP14 predictor groups have a strong correlation, and DP scores ranged from −3.06 to 0.63 (Figures 2E,F). The prediction model with highest DP score, 0.63 for AF2 model 2 (model 427_2) is higher than the highest DP score for any of the NMR conformers, 0.58 (Figure 2E); that is, *some AF2 models fit the NOESY data better than the NMR model itself*.

**FIGURE 3:**
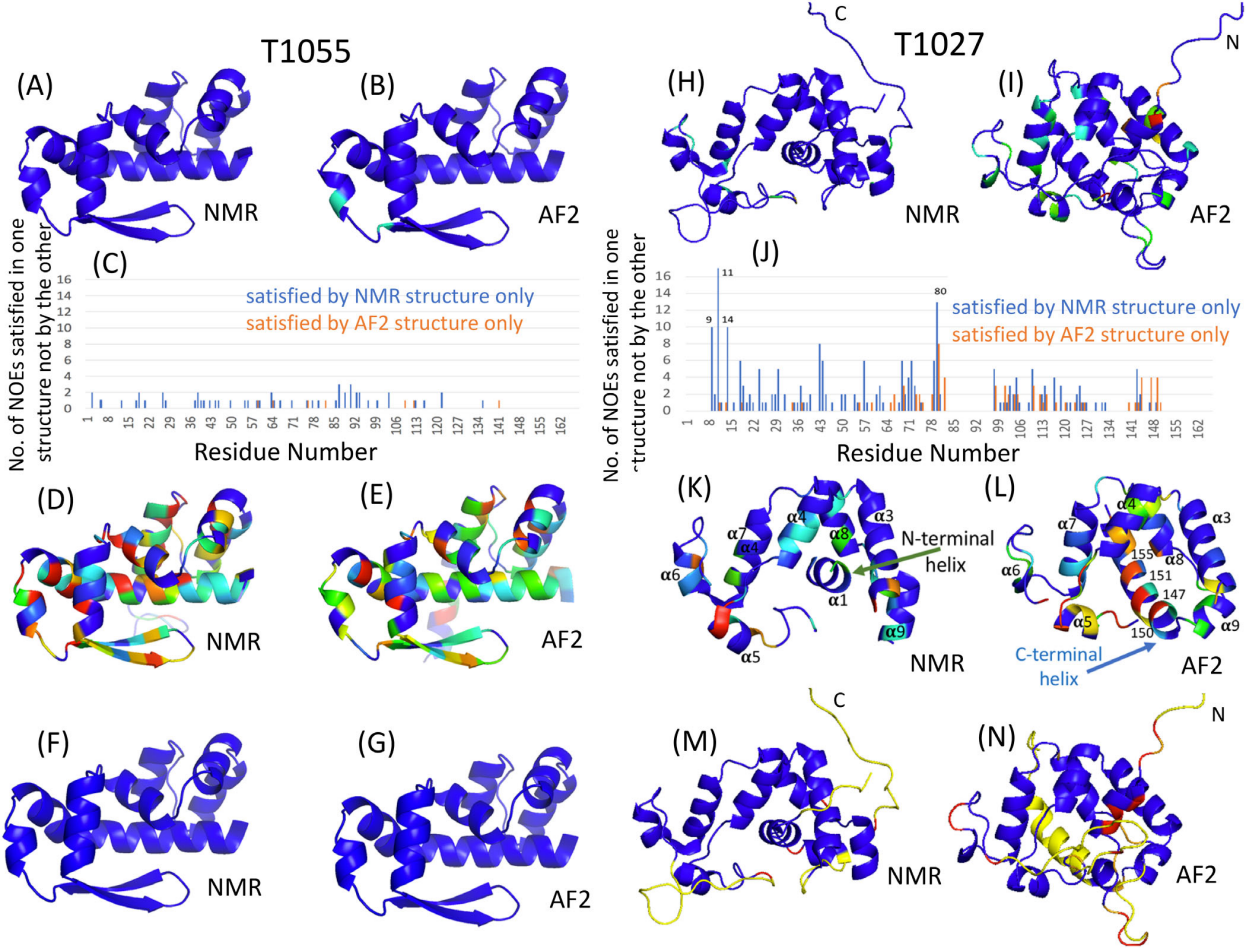
*RPF* and *Talos_N* analysis for CASP14 targets 1055 and 1027. (left) Ensemble Recall analysis for the NMR structure (A) and AF2 model (B) of T1055. Residues with a few NOEs that are assigned and satisfied in the NMR model, but with recall violations for the AF2 models, are colored in light blue in the AF2 model. (C) Plot of number of NOEs that are satisfied in NMR structures but not in AF2 models (blue), or satisfied in AF2 models but not in NMR structures (orange), are plot along the sequence; most NOEs can be explained by both structures. (D,E) Precision analysis for the NMR structure ensemble and AF2 model ensemble of T1055. Residues with modest numbers of Precision violations are colored light blue or green, and those with significant numbers of precision violations are colored yellow, orange and red. (F,G) *Talos_N* analysis for the ensembles of NMR structures and AF2 models of T1055. No significant violations of dihedral angle restraints derived from backbone chemical shift data are observed in any of the NMR structures or AF2 models. (right) Ensemble recall analysis for the NMR structure (H) and AF2 model (I) of T1027. Residues with a NOEs that are assigned and satisfied in the NMR model, but with recall violations for the AF2 models, are colored as outlined in the text on the AF2 model, and vice versa. (J) Plot of number of NOEs that are satisfied in NMR structures but not in AF2 models (blue), or satisfied in AF2 models but not in NMR structures (orange), along the sequence; many NOEs can be explained only by the NMR models. (K,L). Precision analysis for the NMR structure ensemble and AF2 model ensemble of T1027. Residues with modest numbers of Precision violations are colored light blue or green, and those with significant numbers of precision violations are colored yellow, orange and red. (M,N) *Talos_N* analysis for the ensembles of NMR structures and AF2 models of T1027. Residues colored yellow are indicated by chemical shift data to be flexible; residues colored red have backbone conformations in well‐defined regions of the models that are inconsistent with the chemical shift data. In all images, the dark blue color indicates little or no metric violation. In mapping precision violations on the models (e.g., panels K and L) the regions of the structure that are not converged are not shown because precision violations in these regions can arise simply from the conformational variability

RPF DP analysis also provides information about which regions of experimental and prediction models fit to, or violate, the NOESY data. This analysis for target 1055 is summarized on the left side of Figure 3. The recall analysis (NOESY peaks that cannot be explained by the model) indicates that most NOESY peaks are consistent with both the NMR and AF2 models. Overall, the NMR models (*R* = 0.97) have slightly fewer recall violations than the AF2 models (*R* = 0.95–0.96). There are a small number of NOESY peak data that are consistent with the AF2 models, but not the NMR model (Figure 3A,C), and a small number of NOESY peaks that are consistent with the NMR models but not with the AF2 models (Figure 3B,C). The histogram plot (Figure 3C) indicates only 7 NOESY peaks consistent with the AF2 structure, but not the NMR structure, while 54 NOESY peaks are consistent with the NMR structure, but not the AF2 structure.

On the other hand, both the NMR (*P* = 0.74–0.76) and AF2 (*P* = 0.78–0.79) models have significant numbers of precision violations; that is, short distances that are not supported by NOESY peaks. These are distributed throughout the structures (cf., Figure 3D,E). These precision violations arise mostly from sidechain packing that is not fully consistent with the NOESY peak list data. Overall, the AF2 models have much fewer precision violations, consistent with the better *ProCheck* G‐factor (all dihedrals) and *Molprobity* scores, cited above, which indicate more energetically‐consistent core sidechain packing in the AF2 models. These differences may be related to the quality of force fields and energy refinement protocols used in the NMR and AF2 modeling processes.

Finally, we also assessed how well the NMR and AF2 models satisfy backbone dihedral restraints derived from backbone chemical shift data using *Talos_N*.^16^ As *Talos* restraints were used in the NMR structure determination, the NMR‐derived models were expected to be consistent with this analysis. All of the NMR and AF2 models satisfy these chemical shift data (Figure 3F,G). Overall, both the NMR and AF2 models of T1055 fit well to the NOE and chemical shift data, although the smaller number of precision violations (short distances that are not supported by the NMR data) and better *ProCheck* (backbone and sidechain) and *MolProbity* clash scores for the AF2 models indicates they have somewhat more accurate core sidechain packing.

### 3.2 Target T1027: *Gaussia* luciferase (GLuc)

Luciferases are bioluminescent enzymes. CASP14 target T1027 (GLuc) is a 168‐residue luciferase isolated from the marine organism *Gaussia princeps*.^42^ GLuc catalyzes the oxidation of coelenterazine generating a bright blue light, and is attracting interest as a genetically‐encodable reporter protein. Recombinant GLuc for NMR structure determination was expressed in *E. coli* ^43^ and despite its five disulfide bonds, it was refolded into its active form in amounts sufficient for structural analysis using a Solubility Enhancement Peptide tag.^44^ The solution NMR structure of T1027 was determined by Wu et al.^45^ using triple‐resonance NMR and NOESY data (*τ*_m_ = 80 ms), providing 2573 ± 42 NOESY‐derived distance restraints, together with 183 backbone dihedral angle restraints determined from *Talos+* analysis of chemical shift data, 25 hydrogen bond restraints indicated by amide ^1^H/^2^H exchange data, and restraints for three disulfide bonds. *CYANA 3.98* ^46^ was used for both automated NOESY peak assignment and structure generation; no additional energy refinement was done.

The resulting structure (PDB ID 7d2o), reported as an ensemble of 19 conformers, includes 9 α‐helices, and 5 disulfide bonds. The pairing of three disulfide bonds (C59/C120, C65/C77, and C136/C148) were determined unambiguously by the NMR structure. However due to the proximity of Cys residues along the sequence, pairing of the remaining four cysteine (C52, C56, C123, and C127) disulfide pairings could not be unambiguously distinguished from the NMR structures. The C52/C127 pairing is, however, consistent with at least one proteolytic fragment observed in mass spectroscopy, and statistical analysis of Sγ‐Sγ distances across the ensemble of NMR structures strongly suggested C52/C127 and C56/C123 as the most likely pairings.

Structural convergence, proton linewidth, ^15^N relaxation dispersion, and ^1^H‐^15^N heteronuclear NOE (HetNOE) data indicate that GLuc has extensive internal conformational dynamics. Residues 1–9 (N‐terminal segment), 19–35 (helix α2), 82–95 (loop between helices α5 and α6), and 148–168 (C‐terminal segment) exhibit HetNOE values that indicate flexibility. However, some of these segments include strongly conserved residues, and several lines of evidence suggest that some of these regions, particularly the C‐terminal segment, adopt transient structures due to conformational exchange between folded and unfolded states.^45^

We assessed the similarities between these NMR models and CASP14 prediction models, including AF2 models (Figure 2, right). Again, we excluded residue segments for which atomic positions are not well defined in either the ensemble of 19 NMR‐derived conformers or the ensemble of 5 AF2 conformers. Well‐defined regions of the NMR ensemble include residue ranges 10–18, 36–81, and 96–145 (Figure 2G), while for the AF2 structure residues 36–75 and 96–164 are well‐defined (Figure 2H), as defined by *Cyrange*. The pairwise GDT scores between the NMR model with best DP score and the 5 AF2 conformers for the residues whose backbone positions are well defined in both ensembles, that is, for residues 36–75 and 96–145, ranged from 0.66 to 0.67, corresponding to backbone RMSD's of about 4 Å (Figure 2I). The primary differences between the NMR and AF2 structures involve the packing of helices into the core of the protein structure that is formed by the two antiparallel helical bundles (α3, α4, α7, and α8). In the NMR structure, N‐terminal helix α1 is “grabbed” by this bundle, while in the AF2 model helix α1 is replaced in this core by a new helix formed by the C‐terminal segment (which is at least partially disordered in the experimental NMR structure). cf., Figures 2I and 3K,L. This “switch” also reorients helix α2. These helix‐core packing interactions are mutually exclusive. However it is possible that the two forms (i.e., the NMR structure with helix α1 in the core, and the AF2 structure with the C‐terminal region forming a helix and replacing helix α1 in the core) could exist in dynamic equilibrium, consistent with the observed conformational dynamics in both helices α1 and α2 and in the C‐terminal region, described above.

The AF2 model also includes core sidechain conformations that are very similar to those in the solution NMR structure. Particularly notable are the positions and pairing of the five disulfides bonds, which are in good agreement with the experimental structure (Figure 2J), particularly for the four disulfide paired cysteines located in the well‐defined regions of the NMR structure, including the ambiguous C52/C127 and C56/C123 disulfide pairs. There is somewhat less agreement in superimposition for the C136/C148 disulfide pair, that includes residue Cys148 located in a not‐well‐defined (possibly flexible) region of the NMR model. This prediction of correct disulfide pairing and sidechain conformation is quite remarkable considering that no experimental disulfide pairing information was used in the AF2 modeling.

Structure quality statistics for T1027 were analyzed with the *PSVS* software suite.^22^ The resulting structure quality statistics for both the NMR and AF2 model ensembles are summarized in Tables S3 and S4. Both the NMR and AF2 models generally exhibit excellent structure quality scores. The T1027 NMR structure provides a marginally acceptable wwPDB structure validation report (Figure S1); the *ProCheck* (backbone and sidechain) and *MolProbity* Z scores are at the lower end of the normally acceptable range, which probably simply reflects the fact that no specific energy minimization was used in the structure refinement. As was observed for T1055, the AF2 models of T1027 have better *ProCheck* G‐factor (backbone and sidechain) and *Molprobity* clash scores, attributable to more energetically consistent core sidechain packing.

We next assessed how well the NMR and AF2 structures fit to the experimental NMR chemical shift data (bmrb_id 36 288) and NOESY peak list data using the RPF‐DP score. Plots of DP score versus GDT for all CASP14 predictor groups have a strong correlation, with DP scores ranging from −2.02 to 0.58 (Figures 2K,L). The prediction model with highest DP score, 0.58 for AF2 model 4 (model 1027_427_4) is not as high as the DP scores of any of the NMR conformers, 0.64–0.68 (Figure 2K). In this case, the NMR models fit the NOESY data significantly better than any *CASP14* model, including the AF2 models. Although the GDT score between the AF2 models and this NMR structure is lower than for most AF2 predictions, the NMR model is clearly a better fit to the unassigned NOESY data, as the short ^1^H–^1^H distances in the NMR models are more consistent with the NOESY data than those of the AF2 models.

A more detailed RPF DP analysis for T1027 is summarized on the right side of Figure 3. Overall, the NMR models (*R* = 0.89) have less recall violations than the AF2 models (*R* = 0.85–0.86). The recall analysis also documents that there are many NOESY peaks that are consistent with the NMR models but not consistent with the AF2 models (color coded in Figure 3I). Residues with NOESY peaks that are assigned to consistent interactions in the NMR model but not consistent with the AF2 models are color coded on the AF2 model in Figure 3I according to their residue assignment in the NMR model; that is, residues colored light blue (1–3 recall violations), green (4–5 recall violations), orange (6–10 recall violations), or red (> 13 recall violations) indicate NOESY peaks that are not consistent with the AF2 model but have structurally consistent assignments in the NMR model. NOESY peaks that are not consistent with the AF2 model, but assigned in the NMR model, are also indicated with their residue assignments in the NMR model as blue bars in the histogram plot Figure 3J. Hence, the NMR models explain many more NOESY peaks than the AF2 model.

However, there are also some NOESY peaks that are consistent with the AF2 models but not with the NMR models. These residues are colored light blue or green in Figure 3H, and as orange histogram bars in Figure 3J, and include residues 80, 82, and 144–149 in the C‐terminal segment. These NOESY peaks, though inconsistent with the NMR model, could be explained by a low population of conformers similar to the AF2 structure, with a C‐terminal helix interacting with the core in place of the N‐terminal helix.

Both the NMR (*P* = 0.78–0.80) and AF2 (*P* = 0.76–0.78) models of T1027 have a significant number of precision violations. Precision violations are short distances in the model that cannot be explained by any NOESY cross peak. Figure 3K highlights precision violations of the NMR model, located primarily in helices α5 and α6 (Figure 3K). These precision violations may result in part from exchange broadening of resonances in or near these residues, due to conformational dynamics, making the corresponding NOESY cross peaks too weak to observe. In the AF2 models, the precision violations occur mostly where the C‐terminal segment forms a helix that interacts with the core (Figure 3L); that is, this packing interaction is not fully supported by the NOESY data. These missing NOE data expected for a population AF2 conformers in dynamic equilibrium may also be present but attenuated by exchange broadening. Interestingly, however, as some of the short distances resulting from packing the C‐terminal region as a helix into the core, and displacing helix α1, are consistent with some of the NOESY data (Figure 3J, orange bars), this analysis still supports the potential for a small population of conformers in solution with the helical packing predicted by AF2.

Finally, we assessed how well the NMR and AF2 models of T1027 satisfy backbone dihedral restraints derived from backbone chemical shift data using *Talos_N*.^16^ For conformations in fast (or fast‐intermediate) dynamic exchange, the chemical shifts will be population‐weighted averaged (i.e. generally consistent with the dominant conformation), while NOEs may be present for each of the conformations in dynamic equilibrium, with intensities modulated by the corresponding populations. As expected, the NMR models satisfy most of these chemical shift data (Figure 3M). Residues identified by *Talos_N* as “dynamically disordered” (colored in yellow color in Figures 3 M and N) are located mostly in the N‐ and C‐terminal segments or loop regions. Residues whose dihedral angles in the model are inconsistent with backbone chemical shift data are colored red in Figure 3M,N. These are mostly located in the loop regions, and may reflect conformational dynamics in these loops. However, both the conformations of N‐terminal segment and the C‐terminal helical segment of the AF2 models are not supported by these chemical shift data (Figure 3N); if present in solution the predicted C‐terminal helix is populated only to a low level, and is not reflected in the (population‐weight‐averaged) chemical shift data.

### 3.3 Target T1029: Se0862

Biofilms are communities of microorganisms that are enclosed in extracellular polymeric matrices. They provide protection from environmental stresses, and can confer antibiotic resistance. The cyanobacterium *Synechococcus elongatus* encodes a conserved protein Se0862, CASP14 target T1029, that is required for biofilm regulation.^47^ Isotope‐enriched samples of Se0862 were produced by N.Z. and A.L. as a SUMO fusion, which was processed by Ulp1 SUMO protease cleavage to provide the native 125‐residue protein with no non‐native residues. In this work, a chemical‐shift based CS‐Rosetta model was used to guide the NOESY peak assignments, and NOESY peak assignments were restricted to only cross peaks with low assignment ambiguity. The solution structure was determined from 2045 distance restraints, 192 dihedral angle restraints derived from backbone chemical shift data using *Talos‐N*, and 175 RDCs for H^N^–N, H^α^–C^α^, and C^α^–C′ bond vectors^47^ using *Xplor‐NIH*.^48^ The resulting structure is a well‐converged α + β structure with ααββββαα topology. This NMR structure satisfies the NOE‐based distance restraints, and has an acceptable RDC *Q*‐score of 0.173. TALOS chemical‐shift‐based dynamic order parameters S^2^ indicate a generally rigid structure with localized conformational dynamics in surface loops between helices α1 and α2, strands β1 and β2, and strands β3 and β4.^47^

Structure quality statistics for T1029 (PDB ID 6uf2) were analyzed with the *PSVS* software suite,^22^ and the resulting structure quality statistics for both the NMR and AF2 model ensembles are summarized in Tables S5 and S6. The NMR structure exhibits acceptable knowledge‐based structure quality scores. Notably, the *ProCheck* (backbone), *ProCheck* all dihedral (backbone and sidechain), and *MolProbity* Z scores are all > −1.0, typical of good structures.^22^ The wwPDB Structure Validation Report (Figure S1) also does not flag any serious problems with the T1029 NMR structure. Consistent with the observations for the other NMR targets, the AF2 models have even better *ProCheck* (backbone), *ProCheck* (backbone and sidechain) and *MolProbity* Z scores. It should be noted, however, that acceptable values for these metrics are necessary, but not sufficient, for validating the accuracy of a structure, and even models with poor accuracy may have good knowledge‐based structure quality scores.^28^

**FIGURE 4:**
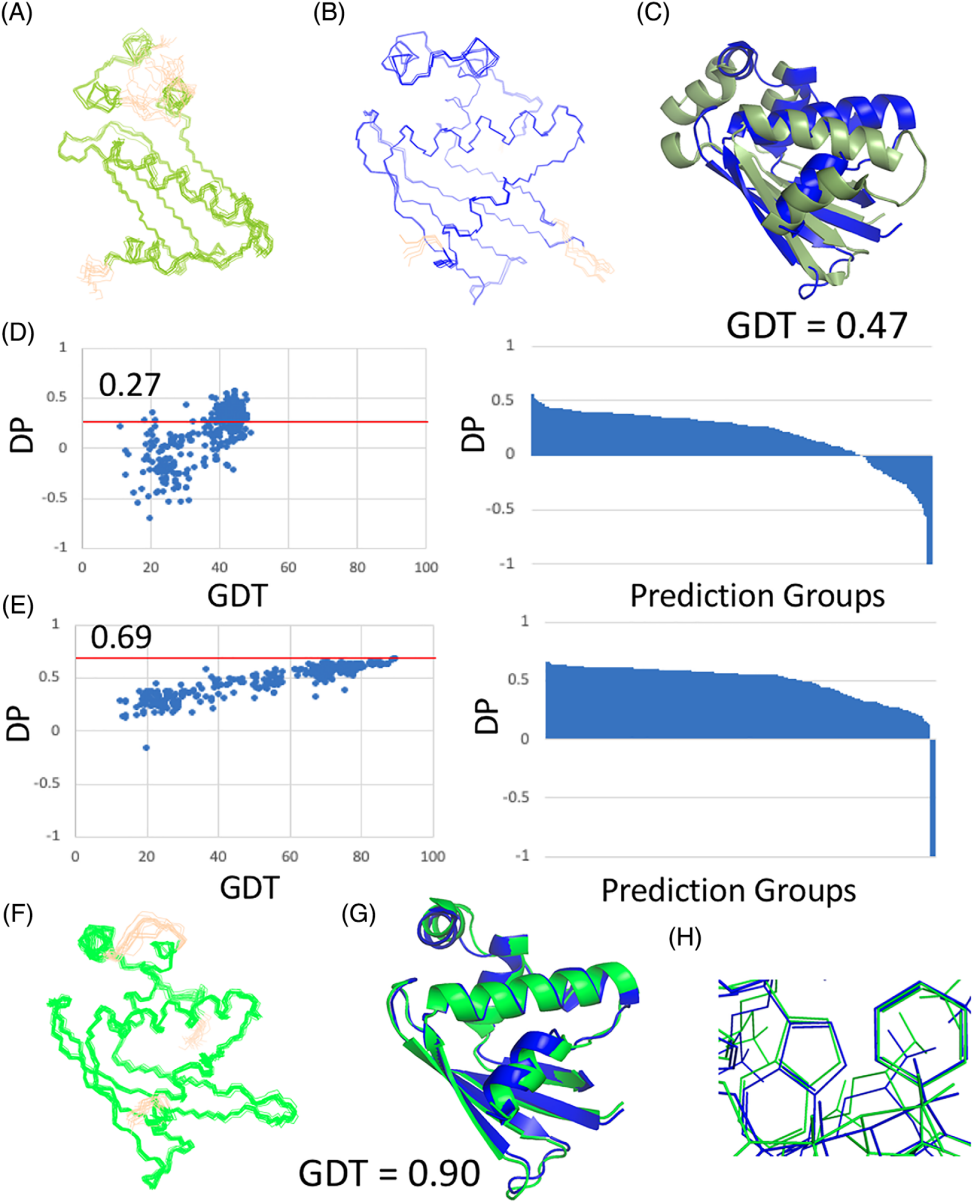
Structural analysis for CASP14 target 1029. Superimposed ensembles for (A) NMR structure (PDB ID 6uf2) (green) and (B) AF2 structure (blue) of T1029. For the NMR structure, residues 3–19 and 29–122 are well‐defined, while for the AF2 structure residues 2‐46 and 53–123 are well‐defined. (C) Comparison of AF2 conformer with highest GDT score (blue) with the representative conformer from the original NMR structure ensembles with best DP score, for residues 3–19, 29–46, and 53–122. (D,E) DP versus GDT scores and DP scores versus predictor group for original NMR structure (D) and revised NMR structure (E). The red horizontal lines in (D) and (E) are drawn at the DP scores of the best scoring conformation from the ensemble of experimental structures. (F) Revised NMR structure (PDB ID 7n82) (green), illustrating the not‐well‐defined segments (brown). Residues 3–20 and 26–123 are well‐defined. (G) Comparison of AF2 conformer with highest GDT score (blue) with the representative conformer from the original NMR structure ensembles with best DP score, for residues 3–19, 29–46 and 53–122. The well‐defined backbone (N, C^α^, C′) atoms are superimposed and both the backbone superimposition and associated core sidechains are illustrated (H). Only residues that are well‐defined in both the original NMR, revised NMR and AF2 structures were included in superimposition and GDT score calculations

We assessed the similarities between NMR and all CASP14 prediction models of T1029 (Figure 4). Well‐defined regions of the NMR ensemble, residue ranges 3–19, and 29–122 (Figure 4A) were identified using *Cyrange*. For the AF2 models, residue ranges 2–46, and 53–123 are well‐defined based on *Cyrange* (Figure 4B), and the pairwise GDT scores between the NMR model with the best DP score and 5 AF2 conformers for residues 3–19, 29–46, 53–122 (i.e., well‐defined in the NMR and AF2 ensemble, and revised NMR ensemble described below), range from 0.46 to 0.47 (Figure 4C), corresponding to a backbone RMSD of ~7 Å. Considering only the common secondary structure elements, the GDT is 0.54–0.55 and backbone RMSD is ~5 Å. The best GDT score for all prediction models is also quite low, GDT = 0.50 for model 1 of prediction group 071 (model 071_1, for residues 3–19, 29–46, 53–122). T1029 is a significant outlier for AF2 and other CASP14 predictions.

We next assessed how well the NMR and CASP14 structures fit to the experimental NOESY peak list data, using the RPF‐DP score.^14^, ^15^ For T1029, the plot of DP score versus GDT for all CASP14 predictor groups has a poor correlation (Figure 4D), and DP scores range from – 1.62 to 0.57 (Figures 2K,L). The highest DP score for all prediction models, 0.57 for model 4 of predictor group 323 (model 323_4), is significantly higher than the range of DP scores obtained for the NMR conformers, 0.19–0.27 (Figure 4D). Indeed, more than 50% of the CASP14 prediction models have DP scores > 0.27, and are a better fit to these NMR data than the NMR structure itself.

### 3.4 Inverse structure determination of T1029

The low DP score for the T1029 NMR model (DP_best = 0.27) is attributable primarily to poor precision scores (P_best = 0.57); that is, there are many short distances in the model that are not explained by the NOESY data. Although a low precision score can result from conformational exchange broadening,^14^, ^15^ T1029 does not exhibit extensive internal dynamics that cause exchange broadening.^47^ This led us to assess the quality of the ^13^C‐ and ^15^N‐edited 3D NOESY peak lists. Because of the strategy of focusing on unambiguously‐assigned NOESY peaks used in the original structure determination process, many peaks present in the NOESY spectra were not included in the original NOESY peak lists nor used in the structure calculations, particularly for the 3D ^13^C‐edited NOESY spectrum. Accordingly, we (N. Z., A. L., Y. J. H., and G. T. M.) carried out a careful repicking of the 3D ^15^N‐ and ^13^C‐edited NOESY data. Due to the relatively low quality of the processed NOESY spectra, automatic peak picking was challenging and resulted in far too many peaks, particularly for the ^13^C‐edited NOESY. In order to guide this peak picking, we then used the recall violations provided by the *RPF* webserver^14^ to further edit these NOESY peak lists by removing peaks with unusual line shapes that are not explained by either the original NMR structure PDB ID 6uf2 nor the AF2 model. The resulting improved NOESY peak lists provided better DP scores for the original NMR structure, of 0.49–0.51, and also higher DP scores for many of the CASP prediction models.

**FIGURE 5:**
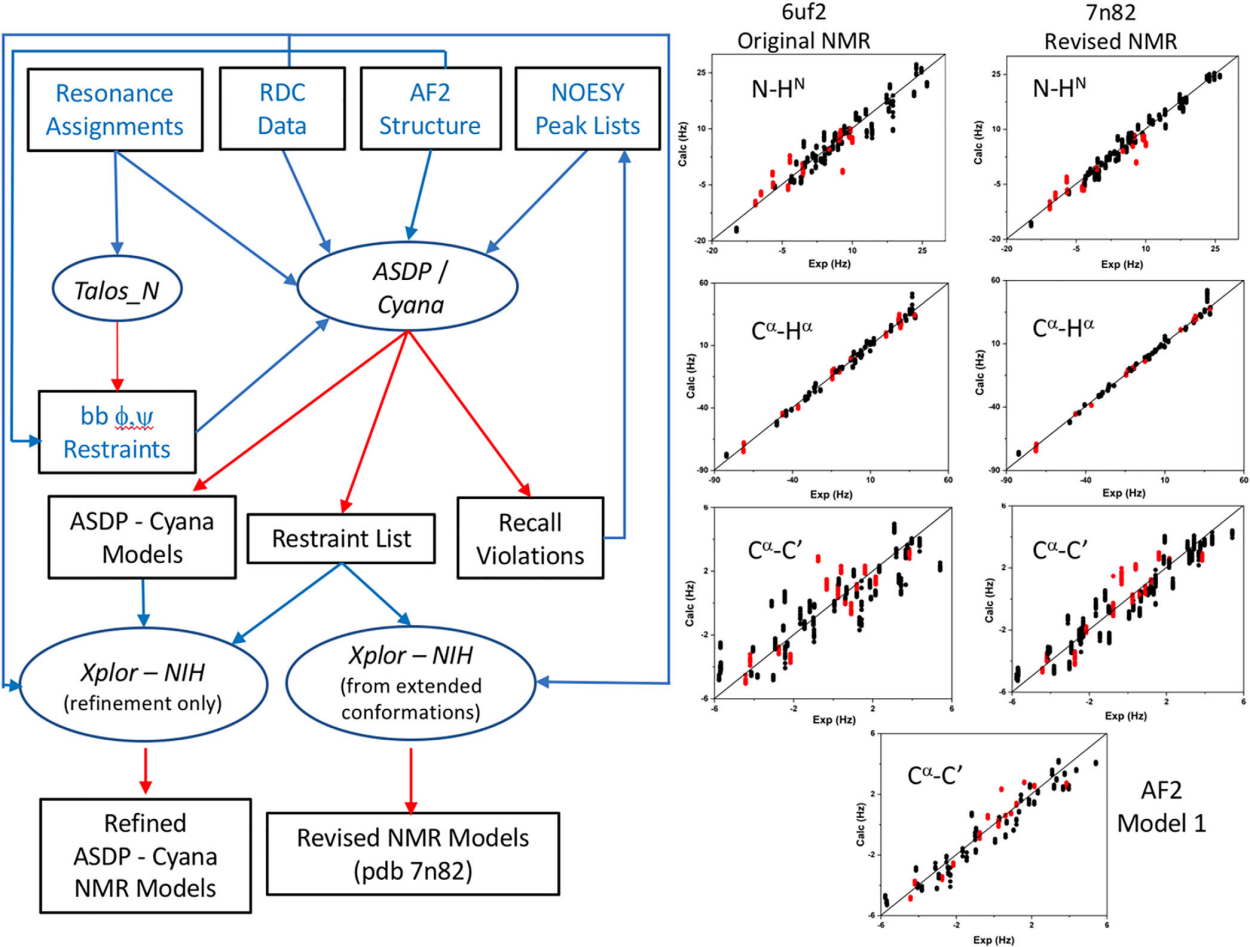
Inverse structure determination. (left) Flow chart of inverse structure determination of T1029 using AF2 model as input. The AF2 models, resonance assignments, *Talos‐N* dihedral restraints, and RDC restraints were combined with the manually‐refined NOESY peak lists and used as input for NOESY peak assignment with the program *ASDP*. The Recall violation list (NOESY peaks not consistent with resulting models) was then used to further guide manual refinement of the NOESY peak list, and the process was reiterated. Blue and red arrows indicate program input and output, respectively. (right) Plots of calculated versus observed RDCs for H^N^–N, H^α^–C^α^, and C^α^–C′ bond vectors for original and revised NMR structures, and RDCs for C^α^–C′ bond vectors for AF2 models

Considering these observations, we (N. Z., A. L., Y. J. H., and G. T. M.) next undertook a refinement of the solution NMR structure of T1029, guided by the AF2 prediction model. This process is outlined on the left side of Figure 5. The resonance assignments, dihedral restraints from *TALOS_N*, and RDC restraints, together with the manually‐refined NOESY peak lists, were used as input for NOESY peak assignment with the program ASDP. However, rather than initializing the ASDP NOESY peak assignment process with an extended or random conformation, the program was initiated with the coordinates of the five AF2 prediction models. Backbone dihedral angle restraints for residues 40, 41, 61, 63, and 123, located in surface loops, that were strongly violated by the AF2 models were also removed from the dihedral restraint list. In this way, the NOESY peak assignment process was intentionally guided by the AF2 prediction models.

In the course of analyzing NOESY peak assignments, *ASDP* uses a structure generation program to produce structural models; in this case, the *Cyana* program was used with the NOESY peak assignments and restraints provided as input to *Cyana* by *ASDP*. The output of *ASDP* also includes assigned NOESY peak lists, distance restraints, and a RFP recall / precision analysis. The recall violation list (NOESY peaks not consistent with resulting models) was then used to further guide manual refinement of the NOESY peak list, and the process was reiterated. The resulting restraints (distance, dihedral, and RDC) were then used as input to *Xplor‐NIH*, using the same protocols used to generate the original NMR structure PDB ID 6uf2. Two structure determination protocols were used with *Xplor‐NIH*: (i) refinement of the *ASDP* ‐ *Cyana* models and (ii) generation of revised NMR models starting from extended conformations. Although both protocols provided acceptable structures, only the results of the second protocol (starting from extended conformations) was selected for deposition and release in the PDB (PDB ID 7n82).

The revised NMR models were analyzed for restraint satisfaction and knowledge‐based structure quality statistics using the *PSVS* program. The knowledge‐based Z scores of *ProCheck* (backbone), *ProCheck* (backbone and sidechain), *ProsaII*, and *MolProbity* for the revised T1029 structure (Table S7) are all significantly better than for the original NMR structure (Table S5), though still a bit lower than those for the AF2 structure (Table S6). The revised NMR models are also a better fit to the RDC data (right side of Figure 5 and Table 1); the Q‐scores for N–H^N^, C^α^–C′, and C^α^–H^α^are all significantly lower (better). In this analysis, we also assessed ANSURR scores.^30^ These are significantly higher (better) for both the *ASDP‐Cyana* NMR models and for the revised NMR structure of T1029 (PDB ID 7n82) than for the original NMR structure (PDB ID 6uf2) (Table 1). The revised NMR structures also have DP scores that are much higher (better) than the original NMR structure, ranging from 0.66 to 0.69, with improved recall and precision statistics (*R* = 0.86–0.87, *P* = 0.75–0.77). Accordingly, the AF2 model was successfully used to guide the analysis of NMR data to produce a revised NMR model with excellent energetics, restraint satisfaction, and a better fit to the NOESY and RDC data than the original NMR structure. Even though the re‐analysis of the T1029 NMR data was guided by the AF2 models, the resulting structures are not identical to the AF2 models, and in fact the DP scores of the revised NMR models are a bit higher than the AF2 models; that is, the revised NMR models are a better fit to the NOESY data than the AF2 models.

**TABLE 1:** RDC data fits and ANSURR scores for target T1029

	<*Q* scores>			<ANSUUR scores>	
Model ensemble	N–H^N^	C^α^–C′	C^α^–H^α^	RMSD	Corr.
Original NMR (6uf2) (10 conformers)	0.154 ± 0.0084	0.021 ± 0.00047	0.159 ± 0.018	48.2 ± 7.7	30.43 ± 5.7
AF2 (5 conformers)	n.d.	0.013 ± 0.00055	n.d.	87.6 ± 2.0	55.2 ± 7.5
ASDP‐Cyana NMR (18 conformers)	0.115 ± 0.0078	0.016 ± 0.00059	0.135 ± 0.013	75.5 ± 7.17	52.9 ± 12.0
Xplor Revised NMR (7n82) (20 conformers)	0.106 ± 0.0028	0.014 ± 0.00057	0.128 ± 0.015	67.9 ± 6.05	53.8 ± 9.2

The revised NMR models (Figure 4F) were then used to reanalyze the DP versus GDT score plot for all CASP14 predictions (Figure 4E). Using the revised NMR model with highest DP score as a reference, the DP versus GDT plot is much more monotonic and linear, as expected for a good quality NOESY peak list and reference model. The prediction models with highest GDT and DP scores were all AF2 models (GDT = 0.89–0.90, DP = 0.66–0.67). These AF2 models also have very good C^α^–C′ RDC scores (Table 1). (N‐H^N^ and C^α^‐H^α^ RDC scores depend on the details of H atom placement, which are not provided in the AF2 model coordinates). The core sidechains in AF2 models also superimpose remarkably well with sidechain conformations in the revised NMR models (Figure 4H).

In order to determine if AF2 had found a lower *Xplor* energy solution not sampled by the NMR analysis, we also assessed the conformational energies of the revised NMR models and AF2 models, for T1027, T1029, and T1055, in the *Xplor* v3.3 force field (without a contribution to the composite energy term from the restraints). This test is complicated by the fact that hydrogen atoms needed to be added to the AF2 models (with *Reduce*). In this analysis, the AF2 models are not as energetically‐favorable as the revised NMR models in the *Xplor* force field. However, these calculations do not properly account for water structure, solvation, dynamics, and other contributions to the free energy, and many of the established knowledge‐based structure quality metrics, such as Ramachandran distributions, *Procheck* backbone and sidechain dihedral angle distributions, and *Molprobity* core sidechain packing scores (with H atoms added), are consistently better for the AF2 structures than for the NMR structures.

### 3.5 NMR guided prediction of an integral membrane protein structure in CASP14

A preliminary solution NMR structure of 238‐residue [^2^H,^13^C, ^15^N‐enriched, ^13^CH_3_ labeled]‐MipA in detergent micelles has been determined using *ASDP* with *Cyana*, followed by refinement with *Rosetta*. The structure is a 10–12 stranded beta‐barrel. The solution NMR structure analysis of MipA is challenging due to extensive exchange broadening in polypeptide segment 43–67, which appears to involve multiple conformations for two strands of the beta‐barrel. The current “best” experimental NMR model has a DP score of 0.54; it is not considered a final structure. Ongoing studies are aimed at properly characterizing these multiple conformational states of MipA, and their relationship to MipA's function.

**FIGURE 6:**
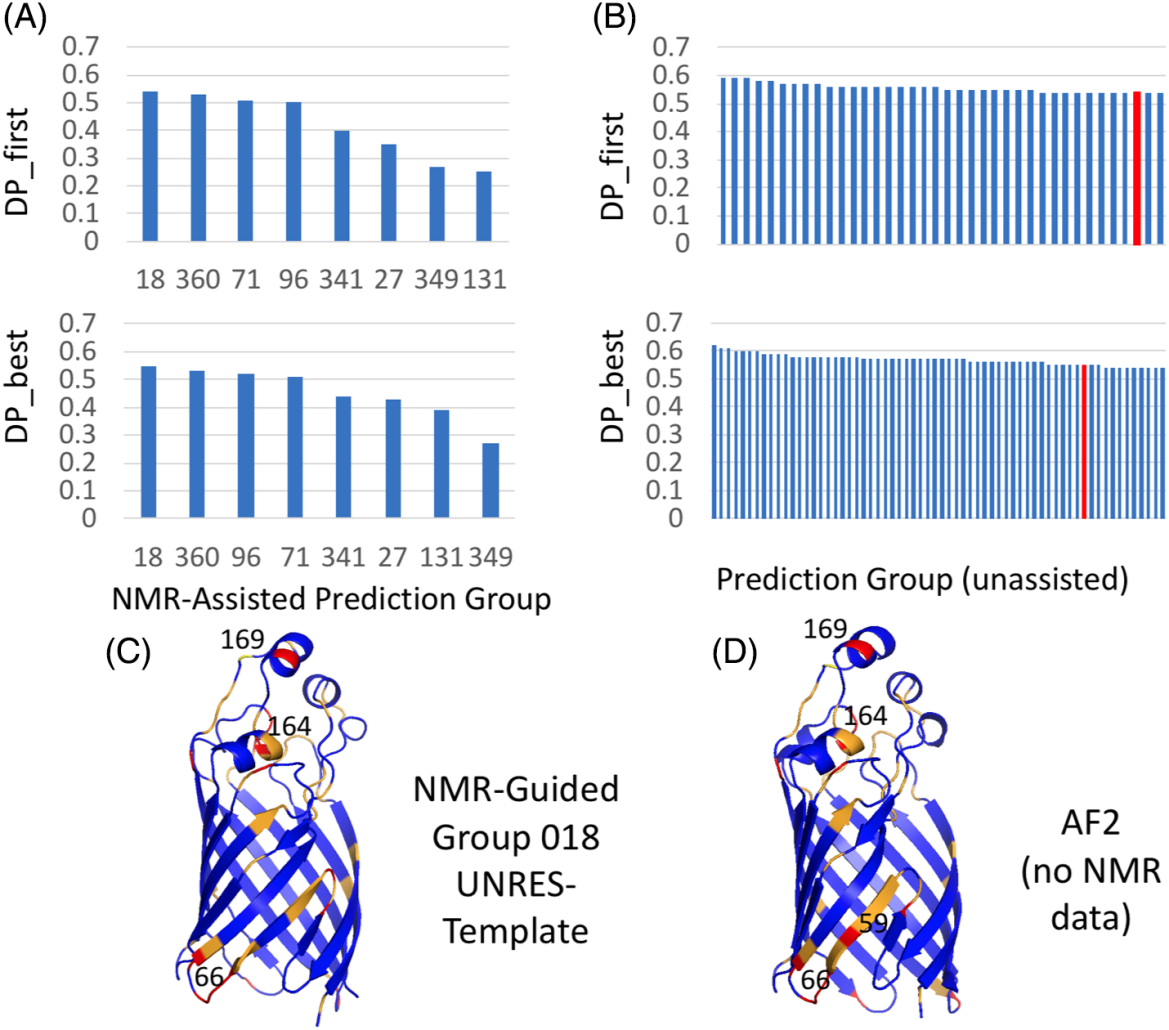
NMR‐assisted prediction of an integral membrane protein. Ranking of (A) NMR assisted and (B) unassisted (pure prediction) CASP models based on the DP score of the predictor‐defined first model (DP_first) or the best scoring model submitted (DP_best). Scores for the AF2 predictor group are highlighted in red among the unassisted prediction groups. (C) NMR‐assisted model and (D) regular prediction (unassisted) model with the best DP scores. The models are colored with information from *TALOS_N*: blue, residues for which backbone conformation is consistent with chemical shift data; red, residues for which backbone conformation is not consistent with chemical shift data; orange, residues with no consensus dihedral angles predicted by *Talos_N*; yellow, residues that chemical shift data indicate to be dynamic. Residues 59, 66, 164, and 169 (red) are labeled as reference points. Residues in segments 52–67 and 162–169, which have backbone conformations that are identified by *Talos_N* as dynamic (yellow), inconsistent (red), or no consensus (orange), but also located in predicted regular secondary structures are considered to be inconsistent with the backbone chemical shift data, and may involve multiple conformations

Since the experimental dynamic solution NMR structure analysis of MipA is still in progress, CASP14 prediction models were assessed only against the NMR NOESY and chemical shift data, using the DP score and *TALOS_N*, rather than against atomic coordinates. In CASP14, eight prediction methods submitted results for “NMR‐assisted prediction” of MipA, in which prediction was assisted by the NOESY‐based ambiguous contact list. A ninth predictor group deposited results in this category, but later informed us that their result did not actually use the Ambiguous Contact Lists derived from NMR data. The relative performance of these eight groups was assessed by DP score for both the top model selected by the submitting group (DP_first) and the best scoring model (DP_best) (Figure 6A). CASP14 prediction groups 018 (UNRES_template), 360 (UNRES), 71 (Kihara Lab) and 96 (UNRES‐contact) all submitted similar beta—barrel structures with DP scores >0.50; the best‐scoring model (N1088TS018_2) has a DP score of 0.55. Analysis of this top‐scoring model against chemical shift data using *TALOS_N* (Figure 6B) showed generally good agreement over most of the structure, except in the polypeptide segment 43–67 for which chemical shift and other NMR data indicate multiple conformations of the local structure. Some residues in the two short α‐helices predicted to form in segment 162–169 also violate the chemical shift data (Figure 6B, residues colored red and yellow). Overall, the top scoring NMR‐assisted prediction models are consistent with one another and in good agreement with the NMR data, except for regions 43–67 (predicted to form two strands of the β‐barrel) and 162–169 (predicted to form two small helices) for which experimental data indicate conformational dynamics.

Next we also assessed all “pure” predictions (i.e., predictions that did not use the NMR—derived ambiguous contact list data) of MipA, using the DP score. Thirty eight top‐scoring prediction groups submitted models with DP_first ≥ 0.54 (Figure 6C) that fit these NMR data better than or equal to the best NMR‐assisted models, and 64 groups with DP_best ≥ 0.54. The best‐scoring models include T1088TS226_5 with DP = 0.62 (Zhang‐TBM), T1088TS024_5 with DP = 0.61 (DeepPotential), T1088TS031_5 with DP = 0.61 (Zhang‐CEthreader), T1088TS328_2 with DP = 0.60 (FoldXpro), T1088TS013_2 with DP = 0.60 (FEIG‐S), T1088TS067_3 with DP = 0.60 (ProQ2), and T1088TS498_3 with DP = 0.60 (VoroMQA‐select). Interestingly, these best‐performing pure prediction groups include (but is not lead by) the DeepMind AF2 group (DP_first = 0.54, and DP_best = 0.55, highlighted by the red histogram bars in Figures 6C). Hence, as was observed in the NMR‐data‐assisted component of CASP13, some advanced pure prediction methods used in CASP14 provided models that fit the NMR data better than traditional or data‐assisted prediction methods that utilize the NMR data itself.

We also tried the inverse structure determination method with MipA, using AF2 models to guide the NOESY assignment process. However, unlike what was observed for target T1029, we did not obtain a complete 12‐stranded β‐barrel structure with this protocol, as the proton resonances that form the key inter strand NOEs needed to form the two missing β‐strands are exchange‐broadened and these NOESY peaks are not present in peak list. The success of the inverse structure determination method is mainly driven by assignment of experimental NOESY cross peaks, rather than being defined directly by the input prediction models.

## 4 DISCUSSION

**FIGURE 7:**
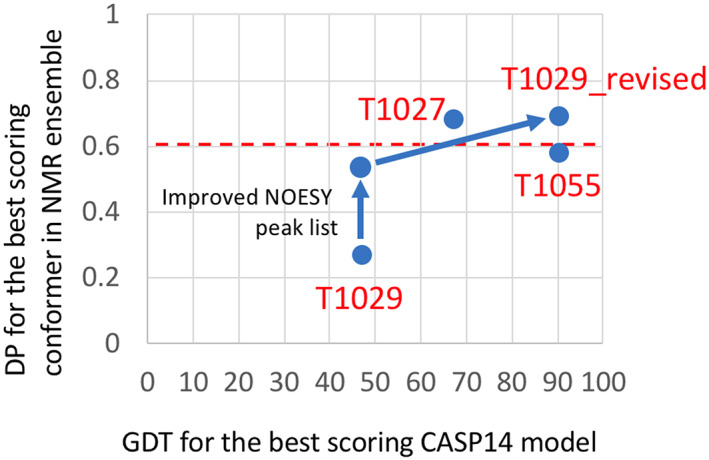
DP and GDT scores for NMR structures in CASP14. Plot of DP score for best‐scoring experimental model versus GDT of best scoring CASP model relative to coordinates of PDB IDs 7d20, 6uf2, 7n82, and 6zyc, for targets T1027, T1029, T1029_revised, and T1055, respectively. The horizontal dashed line is an empirical cutoff for an accurate NMR structure model^14^

In CASP14, the AF2 prediction approach performed remarkably well in predicting 3D structures relative to reference experimental structures determined by X‐ray crystallography and cryoEM, generally providing GDT scores > 0.85.^1^, ^10^ For two of the three targets for which the reference structures were determined by NMR, these scores were generally lower. We initially asked the question whether these lower GDT scores for T1027 and T1029 are due to inaccuracies in the NMR models. For the three NMR structures, we plotted the DP score of best scoring model in the NMR ensemble, a measure of the quality of the NMR structure, against the GDT score for the best‐scoring AF2 model (Figure 7). This analysis suggested that the observed GDT scores for AF2 models of targets T1055 (~ 0.90) and T1027 (~ 0.67) are not attributable to serious problems in the accuracy of these NMR structures. However, the low DP_best score for target T1029 suggested possible inaccuracies in the NMR structure. We investigated this carefully, and refined the NOESY peak list data. Using the improved NOESY peak list, the DP score for the original T1029 structure PDB ID 6uf2 increased significantly, from a range of 0.19 – 0.27 to 0.49 – 0.51. Using the inverse structure determination protocols, the individual conformers of the T1029_revised NMR structure have much higher DP scores (0.66 – 0.69), indicating they are more accurate, than those of the original model. The AF2 models also have much higher GDT scores, 0.89 – 0.90, relative to the revised experimental models T1029_revised (Figure 7).

Our analysis revealed alternate bases for the differences between experimental and prediction models for each CASP14 target. T1055 is a well‐defined, relatively static structure, for which the NOESY and chemical shift data are fit well by either the NMR models deposited in the PDB, or the AF2 models. Interestingly, the AF2 models fit the NMR data a bit better than the experimental structure. These differences are attributable to differences in structure refinement protocols; overall the AF2 and NMR models are nearly identical, with backbone GDT score of 0.90 (corresponding to a backbone RMSD of about 1.3 Å). T1027, on the other hand, is a dynamic structure, for which the experimental data, particularly ^15^N relaxation dispersion data, indicate interconversion between multiple conformational states.^45^ The experimental NMR models are a much better fit to the NOESY and chemical shift data than the AF2 structures; however, some NOEs are not explained by the experimental structure. Features of the distribution of recall and precision violations, and the dynamic NMR data, suggest the potential for a small dynamic population of the AF2‐predicted structure in solution. This interesting hypothesis could be pursued with further data collection and analysis.

For T1029, our analysis revealed that the AF2 model, and other CASP14 prediction models, are a significantly better fit to the NOESY data than the reported NMR structure itself. This observation motivated exploration of a novel method of “inverse structure determination,” in which the predicted AF2 model was used to guide a more complete and accurate analysis of the NMR data. The resulting experimental restraints were then used to generate a revised NMR model ensemble which better fits the NOE, chemical shift, and RDC data. This NMR structure, PDB ID 7n82, has excellent structure validation scores, including *RPF‐DP* and *ANSURR* scores that are significantly better than the original NMR structure.

In computing GDT scores or other superimposition‐based metrics, it is critical to properly exclude those regions of the structure models that are not consistently determined/predicted. In this study, we used the software *Cyrange* ^23^ to distinguish well‐defined from not‐well‐defined regions of the structure, as recommended by the wwPDB Task Force on Protein Structure Validation. Other methods for defining this convention are also useful for this purpose.^49^

The uncertainty in the AF2 models was assessed by the superimpositions shown in Figures 2B,H and Figure 4B. In well‐defined regions the backbone RMSD's across the five models is < 0.5 Å. As discussed in the recent AF2 paper, multiple sequence alignment (MSA) data and co‐variance analysis is part of the input to AF2 predictions, and was used for the four targets shown here. The sensitivity of AF2 structure prediction accuracy to these MSA‐based evolutionary co‐variance information is discussed by Jumper et al.^10^

The available machine learning methods, including AlphaFold2^10^ and RosTTAFold,^50^ are trained on the extensive Protein Data Base (PDB) of protein structures with the assumption that the true structure is a single conformation. Since most of the data in the PDB, and in fact most experimental protein structures, have been provided by X‐ray crystallography, this assumption is relevant. However, in their biological contexts proteins are dynamic and adopt multiple conformational states as required for their thermodynamic stability and functions. While X‐ray crystallography can be used to study protein dynamics, and the various dynamic states of proteins may crystallize separately providing atomic resolution structures for alternative states, other experimental techniques have unique capabilities for characterizing the multiple conformational states of proteins. In particular, both solution NMR and cryo electron microscopy (cryoEM) are especially powerful in identifying and characterizing multiple conformational states of proteins. To date, multiple conformational and dynamic structure prediction has not been a focus in CASP, and is not generally considered in training of machine learning and other protein structure prediction methods. The inaccuracies of AF2 structure predictions for targets T1027 and T1088 may reflect its training to predict a single best structure for the target, rather than a distribution of conformations in dynamic equilibrium.

Another novel result of this work is the sensitivity of the DP versus GDT plots to the correct choice of reference structure for the GDT score calculation. By improving the accuracy of atomic coordinates for target T1029, and using this revised structure as a reference for the GDT calculation (along with improved NOESY peak lists), the correlation between DP and GDT across CASP14 prediction models become much more monotonic and linear. This correlation coefficient is an interesting metric for assessing the correctness of a NMR structural model, a concept which merits further investigation.

In this study, we focus structure validation on RPF‐DP scores,^14^, ^15^ which compare models against unassigned NOESY peak lists, as well as knowledge‐based *Z* scores,^22^ RDC Q scores,^26^ and dihedral angle ranges indicated by chemical shift data.^16^ Generally speaking, distance restraint validation is also an essential metric for NMR structure assessment. The NMR structures deposited in the PDB for the three original targets, T1027, T1029, and T1055, have no significant (> 0.5 Å) restraint violations relative to the deposited restraint lists. The T1029_revised structure also satisfies the restraints used to generate the structure (Table S7). However, distance restraints used in NMR structure determination are derived during an iterative process of NOESY peak assignment, structure generation, and restraint assessment; in some cases NOESY cross peaks may be misassigned, resulting in incorrect restraints, and some restraints may be modified or culled in the process of structure analysis by automated NOESY peak assignment programs. For this reason, our assessment of CASP14 prediction models did not include an extensive analysis of restraint violations relative to the corresponding deposited distance restraint lists; rather we validate models against the NOESY peak lists considering all possible assignments consistent with the chemical shift assignment list.^15^

Another important observation involves the sensitivity of existing structure validation metrics to model inaccuracies. The original T1029 NMR structure has very good structure quality scores, which by standard criteria are acceptable. However, the DP and ANSURR scores suggest some inaccuracies in this ensemble of structures. These problems were not detected by the *PSVS* structure quality score analysis, the wwPDB NMR Structure Validation Report, the RDC *Q* score analysis, the *TALOS_N* analysis, or even by the NOE‐derived restraint violation analysis. These results highlight the weaknesses of these standard NMR structure validation scores for assessing NMR‐derived model accuracy, and the need for using structure versus data scores, like the DP and ANSURR scores, for assessing NMR structure quality.

In CASP14, NMR data were also provided for target T1088, a beta‐type integral membrane porin protein, used by several CASP14 predictor groups to generate NMR‐guided prediction models. Most groups involved in this exercise generated similar beta‐barrel models, with good agreement with the experimental data. However, as was also observed in CASP13,^13^ some regular prediction groups, which did not use the NMR data, generated models for T1088 which better fit the NMR data than the NMR‐guided methods. In all cases, the most severe discrepancies between the predicted models and NMR data are in the segment 43–67 for which NMR data indicate intermediate‐exchange conformational dynamics. These results demonstrate the power of the most advanced current modeling methods to predict structures of small proteins with accuracies rivaling solution NMR structures. However, they also illustrate, again, the shortcomings of prediction methods to identify regions of conformational dynamics and to reliably model alternative conformational states, and suggests the need to validate prediction models against experimental data characterizing conformational dynamics.

The CASP14 blind protein structure prediction results have provided the opportunity to assess the potential for using predicted protein structures to guide experimental NMR data analysis. This goal appears to have been successfully achieved using current best methods of protein structure prediction, for proteins of up to about 200 residues. The best prediction results (e.g., AF2 models) generally fit to the experimental NMR data as well (or better) than experimental structures generated from these same data using conventional approaches. Specifically, in two of the three cases studied here (T1055 and T1029), the AF2 models match the experimental data as well or better than structures generated by conventional NMR structure determination methods.

Considering the results with more than 90 protein targets,^1^ the accuracy of structures predicted by *AlphaFold2* appear to be generally sufficient to provide reliable guidance to NMR data analysis. Several other structure prediction methods applied in CASP14 also achieved nearly this level of model accuracy for relatively static structures. The availability of source code for *AlphaFold2*,^10^ *RosTTAFold*,^50^ and other successful co‐variance and machine learning methods strongly motivates future efforts to explore using these methods to guide NMR data analysis. There is potential to use predicted models not only to guide structure analysis, as was done here, but to provide a complete analysis of both resonance assignments and 3D structures. Accurate models provided by methods like *AlphaFold2* ^10^ and *RosTTAFold* ^50^ open the potential of complete structure determination of small, relatively rigid protein structures from a single NOESY spectrum; for example, from a single simultaneous ^13^C,^15^N‐resolved NOESY spectrum. However, care must be exercised in using prediction models to interpret such experimental data, as was observed for T1029 using a *CS‐Rosetta* structure to guide the analysis of the original T1029 structure.^47^ For example, when there are significant conformational dynamics of the target protein structure, like targets T1027 and T1088, the prediction methods used in CASP14 cannot yet accurately describe these conformational distributions. For such dynamic structures, current prediction methods have limited value in guiding the data analysis, and might in fact misguide the structure analysis process. Methods for predicting chemical shift assignments from models are also not yet sufficiently accurate. Moreover, while structure prediction for less dynamic structures is more reliable, efforts in “inverse structure determination” are susceptible to any errors in the predicted structure that are not contraindicated by the data, and must be carefully cross validated by multiple model versus data structure quality assessment scores including the RPF‐DP score, RDC Q score, and ANSURR score.

## CONFLICT OF INTEREST

The authors declare that there is no conflict of interest. G.T.M. is a founder of Nexomics Biosciences, Inc. and G.L. is Chief Scientific Officer of Nexomics, Biosciences, Inc. These roles do not represent a conflict of interest for this study.

### PEER REVIEW

The peer review history for this article is available at https://publons.com/publon/10.1002/prot.26246.

## Supporting information


**Tables S1** **–S7**. PSVS structure quality reports for experimental NMR and predicted AF2 models.
**Table S8**. Top scoring regular predictor groups for T1088, with DP_best ≥0.54
**Figure S1**. wwPDB NMR structure quality summaries for experimental NMR and predicted AF2 models.

## Data Availability

The data that support the findings of this study are available from the corresponding author upon reasonable request.
