# Supplementary material for: Assessment of prediction methods for protein structures determined by NMR in CASP14: Impact of AlphaFold2
Source: Proteins. 2021 Oct 19;89(12):1959–76. doi: 10.1002/prot.26246 (PMC8616817; doi:10.1002/prot.26246)
Supplement: Supplementary file 1 — Tables S1 –S7. PSVS structure quality reports for experimental NMR and predicted AF2 models. Table S8. Top scoring regular predictor groups for T1088, with DP_best ≥0.54 Figure S1. wwPDB NMR structure quality summaries for experimental NMR and predicted AF2 models. [file PROT-89-1959-s001.pdf]

Supplementary Methods

Mip A Sample Preparation

Protein expression. A codon-optimized ACA-less *mipA* gene from *Klebsiella pneumoniae* was synthesized (Genscript) and cloned into pColdII vector [1, 2] for efficient isotope enrichment in condensed phase. BL21(DE3)*ΔhisB* harboring *pACYCmazF(ΔH)* and *pCold2-mipA without signal peptide or pCold2-yfaZ without signal peptide* was grown in 3L of M9-glucose supplemented with His (20 mg / L) and Amp (100 mg / L) and Cm (25 mg / L). When $O.D_{600}$ reached around 0.5, the culture was cooled down on ice for 5 min, followed by incubation at 16 °C for 45 min. The cells were collected and washed by 1xM9 H_2O for twice, and resuspended into 150 ml of M9-glucose supplemented with Amp (100 mg / L) and Cm (25 mg / L). MazF(ΔH) was induced by the addition of 1 mM IPTG. After incubating at 16 °C for 2hr, the cells were washed twice by 1x M9 D_2O, and resuspended into 300 ml M9 D_2O d7-glucose supplemented with 1g / L ^{15}N $(NH_4)_2SO_4$, 100 mg / L α-ketoisovaleric acid, 50 mg / L α-ketobutyric acid 100 mg / L , and ^{15}N-^{13}C- Ala, and 1 mM IPTG. The culture was further incubated for overnight. The next day, the culture was collected and stored at -20 °C until further use.

Protein purification: The cells were resuspended in 20 ml of 20 mM Tris-HCl (pH 8.0) and cells were disrupted by French press at 16,000 psi. After centrifugation, the unbroken cells were removed by resuspending cells in 10 ml of 20 mM Tris-HCl (pH8), followed by sonication. This process was repeated for 2 more times. Finally, the pellet was resuspended into 20 mL binding buffer (50 mM Na-phosphate, pH 7.5, 300 mM NaCl and 20 mM imidazole, 8M urea). The solution was centrifuged at 40,000 rpm for 1 hr. The supernatant was incubated with Ni-resin

for 1 hr. The resin was washed by binding buffer and eluted with elution buffer (50 mM Na-phosphate, pH 7.5, 300 mM NaCl, and 300 mM imidazole, 8M urea). After purification, the eluted fraction was concentrated and purity was checked by SDS-PAGE followed by Coomassie Blue staining. After confirming the purity of the protein (> 95% homogeneous), the protein was concentrated to 1 mM. The general yields were 20 mg from 300 ml condensed culture.

Protein refolding: One 1 ml of concentrated protein (about 1 mM) was diluted into 9 ml buffer A (20 mM K-phosphate, pH 6.5, containing 0.5 M NaCl, 0.5 M Arg, 0.1% d_{37}-DPC). After concentrating the protein to 1 ml, 9 ml of buffer A was added and concentrated to 1 ml again. Finally, buffer was exchanged with 9 ml buffer B (20 mM K-phosphate, pH 6.5, 0.2 M NaCl, 50 mM M Arg, 0.1% d_{37}-DPC) twice, and concentrated to 250 µl. The protein concentration was assessed by SDS-PAGE using BSA as a standard.

MipA NMR Data Collection and Processing

All NMR spectra were recorded at 45 ºC. NMR data were collected on Bruker AVANCE 600 MHz or 800 MHz spectrometers using cryogenic NMR probes, processed using the program *NMRPipe* [3], and analyzed using the programs *SPARKY* [4] and *XEASY* [5]. Spectra were referenced to external DSS. Backbone resonance assignments were determined using standard ^{2}H-decoupled triple resonance experiments, including NUS-trHNCO, trHNCA, trHNCACB, trHNcoCACB, NUS-trHNcoCA, NUS-trHNcaCO, tr-^{15}N-edit -NOESY. Sequence-specific backbone sidechain methyl resonance assignments were determined from ^{15}N-edited NOESY experiments. Resonance assignments were determined using *AutoAssign* software [6, 7] together with interactive

manual analysis. NMR data collection included simultaneous ^{15}N,^{13}C-edited 3D NOESY and ^{15}N-edited 3D NOESY, both recorded with mixing time τ_m = 300 ms. NOESY spectra were peak-picked manually.

Ambiguous Contact Lists for MipA. NMR data for MipA was provided to CASP14 predictor groups in the form of Ambiguous Contact Lists, prepared as described previously [8] by analyzing the experimental NOESY peak lists together with the corresponding resonance assignment lists using the Cycle 0 module of the program *ASDP* {Huang, 2006 #37}. For each NOESY peak, the output of *ASDP* Cycle 0 provided all possible proton pair assignments within the defined resonance frequency match tolerances of 0.03 ppm, 0.30 ppm, and 0.30 ppm for ^{1}H, ^{15}N, and 13C, respectively. Additional assignment methods of ASDP Cycle 0, such as contact map and secondary structure guided NOESY peak assignments{Huang, 2006 #37} , were not used for this CASP14 experiment. In practice, each NOESY peak is assigned to a set of ambiguous ^{1}H-^{1}H pair assignments whose chemical shifts are compatible with the resonance frequencies associated with the 3D NOESY peak. In the absence of experimental errors, at least one of these ^{1}H-^{1}H pairs should correspond to a short-distance interproton interaction that is consistent with the native protein structure. However, NOESY peaks that arise from unassigned resonances, as well as random noise peaks, will provide a set of ambiguous contacts, or possibly even a unique assignment, none of which are consistent with the native structure. The resulting Ambiguous Contact List for MipA was provided to CASP predictors.

Supplementary References

1. Schneider WM, Inouye M, Montelione GT, Roth MJ. Independently inducible system of gene expression for condensed single protein production (cSPP) suitable for high efficiency isotope enrichment. J Struct Funct Genomics. 2009; 10:219-225. https://doi.org/10.1007/s10969-009-9067-x
2. Suzuki M, Zhang J, Liu M, Woychik NA, Inouye M. Single protein production in living cells facilitated by an mRNA interferase. Molecular cell. 2005; 18:253-261.
3. Delaglio F, Grzesiek S, Vuister GW, Zhu G, Pfeifer J, Bax A. NMRPipe: a multidimensional spectral processing system based on UNIX pipes. J Biomol NMR. 1995; 6:277-293.
4. Goddard TD, Kneller DG. Sparky 3. San Francisco, CA: University of California; 2000.
5. Bartels C, Xia TH, Billeter M, Guntert P, Wuthrich K. The program XEASY for computer-supported NMR spectral analysis of biological macromolecules. Journal of biomolecular NMR. 1995; 6:1-10. https://doi.org/10.1007/BF00417486
6. Zimmerman DE, Kulikowski CA, Huang Y, Feng W, Tashiro M, Shimotakahara S, Chien C, Powers R, Montelione GT. Automated analysis of protein NMR assignments using methods from artificial intelligence. J Mol Biol. 1997; 269:592-610.
7. Moseley HN, Monleon D, Montelione GT. Automatic determination of protein backbone resonance assignments from triple resonance nuclear magnetic resonance data. Methods Enzymol. 2001; 339:91-108.
8. Sala D, Huang YJ, Cole CA, Snyder DA, Liu G, Ishida Y, Swapna GVT, Brock KP, Sander C, Fidelis K, Kryshtafovych A, Inouye M, et al. Protein structure prediction assisted with sparse NMR data in CASP13. Proteins. 2019; 87:1315-1332. https://doi.org/10.1002/prot.25837

Supplementary Table S1: PSVS Report for T1055 - NMR Structure

Analyses performed for well-defined residues.

Number of structures	20		
<u>RMSD Values</u>			
	all	ordered[e]	selected[f]
All backbone atoms	6.6 Å	2.7 Å	2.7 Å
All heavy atoms	7.2 Å	3.0 Å	3.0 Å
Structure Quality Factors - overall statistics			
	Mean score	SD	Z-score [g]
Procheck G-factor [e] (phi / psi only)	-0.04		0.16
Procheck G-factor [e] (all dihedral angles)	-0.26		-1.54
Verify3D	0.18	0.0165	-4.49
ProsaII (-ve)	0.63	0.0370	-0.08
MolProbity clashscore	50.22	3.0388	-7.09
Ramachandran Plot Summary from Procheck [f]			
Most favoured regions	89.1%		
Additionally allowed regions	10.1%		
Generously allowed regions	0.7%		
Disallowed regions	0.0%		
Ramachandran Plot Statistics from Richardson's lab			
Most favoured regions	96.3%		
Allowed regions	3.7%		
Disallowed regions	0%		

[e] Residues with sum of phi and psi order parameters > 1.8

Ordered residue ranges: 4A-123A,137A-139A

[f] Residues selected based on: dihedral angle order parameter, with S(phi)+S(psi)>=1.8

Selected residue ranges: 4A-123A,137A-139A

[g] With respect to mean and standard deviation for for a set of 252 X-ray structures < 500 residues, of resolution <= 1.80 Å, R-factor <= 0.25 and R-free <= 0.28; a positive value indicates a 'better' score

Generated using PSVS 1.5

Supplementary Table S2: PSVS Report for T1055 - AF2 Structure

Analyses performed for well-defined residues.

Number of structures	5		
RMSD Values			
	all	ordered[e]	selected[f]
All backbone atoms	2.2 Å	0.8 Å	0.8 Å
All heavy atoms	2.3 Å	1.1 Å	1.1 Å
Structure Quality Factors - overall statistics			
	Mean score	SD	Z-score [g]
Procheck G-factor [e] (phi / psi only)	0.41	N/A	1.93
Procheck G-factor [e] (all dihedral angles)	0.42	N/A	2.48
Verify3D	0.18	0.0182	-4.49
ProsaII (-ve)	0.84	0.0219	0.79
MolProbity clashscore	2.58	0.6081	1.08
Ramachandran Plot Summary from Procheck [f]			
Most favoured regions	97.7%		
Additionally allowed regions	2.3%		
Generously allowed regions	0.0%		
Disallowed regions	0.0%		
Ramachandran Plot Statistics from Richardson's lab			
Most favoured regions	99.5%		
Allowed regions	0.5%		
Disallowed regions	0%		

[e] Residues with sum of phi and psi order parameters > 1.8

Ordered residue ranges: 14A-127A,135A-146A

[f] Residues selected based on: dihedral angle order parameter, with S(phi)+S(psi)>=1.8

Selected residue ranges: 14A-127A,135A-146A

[g] With respect to mean and standard deviation for for a set of 252 X-ray structures < 500 residues, of resolution <= 1.80 Å, R-factor <= 0.25 and R-free <= 0.28; a positive value indicates a 'better' score

Generated using PSVS 1.5

Supplementary Table S3: PSVS Report for T1027 - NMR Structure

Analyses performed for well-defined residues.

Number of structures used	19		
RMSD Values			
	all	ordered[e]	selected[f]
All backbone atoms	7.4 Å	1.4 Å	1.4 Å
All heavy atoms	7.2 Å	1.7 Å	1.7 Å
Structure Quality Factors - overall statistics			
	Mean score	SD	Z-score [g]
Procheck G-factor [e] (phi / psi only)	-0.04		0.16
Procheck G-factor [e] (all dihedral angles)	-0.52		-3.08
Verify3D	0.10	0.0133	-5.78
ProsaII (-ve)	0.53	0.0497	-0.50
MolProbity clashscore	23.66	1.6608	-2.53
Ramachandran Plot Summary from Procheck [f]			
Most favoured regions	91.0%		
Additionally allowed regions	9.0%		
Generously allowed regions	0.1%		
Disallowed regions	0.0%		
Ramachandran Plot Statistics from Richardson's lab			
Most favoured regions	95.5%		
Allowed regions	4.5%		
Disallowed regions	0%		

[e] Residues with sum of phi and psi order parameters > 1.8

Ordered residue ranges: 8A-18A,23A-27A,35A-78A,97A-134A,137A-144A

[f] Residues selected based on: dihedral angle order parameter, with S(phi)+S(psi)>=1.8

Selected residue ranges: 8A-18A,23A-27A,35A-78A,97A-134A,137A-144A

[g] With respect to mean and standard deviation for for a set of 252 X-ray structures < 500 residues, of resolution <= 1.80 Å, R-factor <= 0.25 and R-free <= 0.28; a positive value indicates a 'better' score

Generated using PSVS 1.5

Supplementary Table S4: PSVS Report for T1027 - AF2 Structure

Analyses performed for well-defined residues.

Number of structures used	5		
<u>RMSD Values</u>			
	all	ordered[e]	selected[f]
All backbone atoms	2.7 Å	1.4 Å	1.4 Å
All heavy atoms	2.9 Å	1.6 Å	1.6 Å
Structure Quality Factors - overall statistics			
	Mean score	SD	Z-score [g]
Procheck G-factor [e] (phi / psi only)	-0.03		0.20
Procheck G-factor [e] (all dihedral angles)	0.13		0.77
Verify3D	0.14	0.0114	-5.14
ProsaII (-ve)	0.71	0.0485	0.25
MolProbity clashscore	7.83	4.8170	0.18
Ramachandran Plot Summary from Procheck [f]			
Most favoured regions	88.1%		
Additionally allowed regions	11.6%		
Generously allowed regions	0.3%		
Disallowed regions	0.0%		
Ramachandran Plot Statistics from Richardson's lab			
Most favoured regions	97.6%		
Allowed regions	2%		
Disallowed regions	0.4%		

[e] Residues with sum of phi and psi order parameters > 1.8

Ordered residue ranges: 2A-4A,8A-13A,34A-164A

[f] Residues selected based on: dihedral angle order parameter, with S(phi)+S(psi)>=1.8

Selected residue ranges: 2A-4A,8A-13A,34A-164A

[g] With respect to mean and standard deviation for for a set of 252 X-ray structures < 500 residues, of resolution <= 1.80 Å, R-factor <= 0.25 and R-free <= 0.28; a positive value indicates a 'better' score

Generated using PSVS 1.5

Supplementary Table S5: PSVS Report for T1029 - original NMR Structure

Analyses performed for well-defined residues.

Number of structures used	10		
<u>RMSD Values</u>			
	all	ordered[e]	selected[f]
All backbone atoms	0.7 Å	0.4 Å	0.4 Å
All heavy atoms	1.3 Å	1.0 Å	1.0 Å
Structure Quality Factors - overall statistics			
	Mean score	SD	Z-score [g]
Procheck G-factor [e] (phi / psi only)	-0.12		-0.16
Procheck G-factor [e] (all dihedral angles)	-0.15		-0.89
Verify3D	0.14	0.0202	-5.14
ProsaII (-ve)	0.27	0.0231	-1.57
MolProbity clashscore	4.25	0.7169	0.80
Ramachandran Plot Summary from Procheck [f]			
Most favoured regions	91.1%		
Additionally allowed regions	7.4%		
Generously allowed regions	1.5%		
Disallowed regions	0.0%		
Ramachandran Plot Statistics from Richardson's lab			
Most favoured regions	96.7%		
Allowed regions	3.3%		
Disallowed regions	0%		

[e] Residues with sum of phi and psi order parameters > 1.8

Ordered residue ranges: 2A-20A,28A-124A

[f] Residues selected based on: dihedral angle order parameter, with S(phi)+S(psi)>=1.8

Selected residue ranges: 2A-20A,28A-124A

[g] With respect to mean and standard deviation for for a set of 252 X-ray structures < 500 residues, of resolution <= 1.80 Å, R-factor <= 0.25 and R-free <= 0.28; a positive value indicates a 'better' score

Generated using PSVS 1.5

Supplementary Table S6: PSVS Report for T1027 - AF2 Structure

Analyses performed for well-defined residues.

Number of structures used	5		
RMSD Values			
	all	ordered[e]	selected[f]
All backbone atoms	0.2 Å	0.2 Å	0.2 Å
All heavy atoms	0.4 Å	0.4 Å	0.4 Å
Structure Quality Factors - overall statistics			
	Mean score	SD	Z-score [g]
Procheck G-factor [e] (phi / psi only)	0.08		0.63
Procheck G-factor [e] (all dihedral angles)	0.21		1.24
Verify3D	0.22	0.0084	-3.85
ProsaII (-ve)	0.66	0.0344	0.04
MolProbity clashscore	1.30	0.8367	1.30
Ramachandran Plot Summary from Procheck [f]			
Most favoured regions	95.9%		
Additionally allowed regions	3.6%		
Generously allowed regions	0.6%		
Disallowed regions	0.0%		
Ramachandran Plot Statistics from Richardson's lab			
Most favoured regions	98.2%		
Allowed regions	1.8%		
Disallowed regions	0%		

[e] Residues with sum of phi and psi order parameters > 1.8

Ordered residue ranges: 2A-124A
[f] Residues selected based on: dihedral angle order parameter, with S(phi)+S(psi)>=1.8

Selected residue ranges: 2A-124A

[g] With respect to mean and standard deviation for for a set of 252 X-ray structures < 500 residues, of resolution <= 1.80 Å, R-factor <= 0.25 and R-free <= 0.28; a positive value indicates a 'better' score

Generated using PSVS 1.5

Supplementary Table S7: PSVS Report for T1027 - Revised NMR Structure - including restraint violation analysis

Summary of conformationally-restricting experimental restraints [a]			
NOE-based distance constraints:			
Total	1370		
intra-residue [i = j]	273		
sequential [| i - j | = 1]	451		
medium range [1 < | i - j | < 5]	279		
long range [| i - j | ≥ 5]	367		
NOE restraints per restrained residue [b]	11.1		
Hydrogen bond restraints:			
Total	42		
long range [| i - j | ≥ 5]	20		
Dihedral-angle restraints:	410		
Total number of restricting restraints [b]	1822		
Total number of restricting restraints per restrained residue [b]	14.8		
Restricting long-range restraints per restrained residue [b]	3.1		
Number of structures used	20		
Residual restraint violations [a,c]			
Distance violations / structure			
0.1 - 0.2 Å	30.7		
0.2 - 0.5 Å	26.05		
> 0.5 Å	2.8		
RMS of distance violation / restraint	0.07 Å		
Maximum distance violation [d]	0.82 Å		
Dihedral angle violations / structure			
1 - 10 °	4.6		
> 10 °	0		
RMS of dihedral angle violation / restraint	0.19 °		
Maximum dihedral angle violation [d] RMSD	3.20 °		
Values			
	all	ordered[e]	selected[f]
All backbone atoms	0.4 Å	0.3 Å	0.3 Å
All heavy atoms	0.8 Å	0.7 Å	0.7 Å

PSVS Software Environment

	Mean score	SD	Z-score [g]
Procheck G-factor [e] (phi / psi only)	0.05		0.51
Procheck G-factor [e] (all dihedral angles)	0.03		0.18
Verify3D	0.18	0.0103	-4.49
ProsaII (-ve)	0.52	0.0279	-0.54
MolProbity clashscore	1.48	1.3061	1.27
Ramachandran Plot Summary from Procheck [f]			
Most favoured regions	95.2%		
Additionally allowed regions	4.1%		
Generously allowed regions	0.4%		
Disallowed regions	0.2%		
Ramachandran Plot Statistics from Richardson's lab			
Most favoured regions	98.8%		
Allowed regions	1.1%		
Disallowed regions	0%		

[a] Analysed for residues 1 to 125
[b] There are 123 residues with conformationally restricting constraints
[c] Calculated for all restraints for the given residues, using sum over r^-6
[d] Largest constraint violation among all the reported structures
[e] Residues with sum of phi and psi order parameters > 1.8

Ordered residue ranges: 2A-20A,25A-124A
[f] Residues selected based on: dihedral angle order parameter, with S(phi)+S(psi)>=1.8

Selected residue ranges: 2A-20A,25A-124A

[g] With respect to mean and standard deviation for for a set of 252 X-ray structures < 500 residues, of resolution <= 1.80 Å, R-factor <= 0.25 and R-free <= 0.28; a positive value indicates a 'better' score

Generated using PSVS 1.5

Supplementary Table S8. Top scoring regular predictor groups for T1088 MipA, with DP_best ≥ 0.54

Group	DP_First	DP_Best
226	0.47	0.62
24	0.54	0.61
31	0.52	0.61
328	0.55	0.6
13	0.5	0.6
67	0.5	0.6
498	0.5	0.6
125	0.59	0.59
453	0.59	0.59
487	0.59	0.59
435	0.49	0.59
187	0.58	0.58
253	0.58	0.58
319	0.57	0.58
101	0.56	0.58
293	0.56	0.58
403	0.56	0.58
32	0.55	0.58
26	0.51	0.58
198	0.51	0.58
222	0.51	0.58
75	0.57	0.57
343	0.57	0.57
351	0.57	0.57
254	0.56	0.57
323	0.56	0.57
334	0.56	0.57
473	0.56	0.57
379	0.55	0.57
15	0.54	0.57
39	0.54	0.57
472	0.54	0.57
62	0.52	0.57
183	0.52	0.57
314	0.5	0.57
375	0.49	0.57
252	0.56	0.56
324	0.56	0.56

326	0.56	0.56
129	0.55	0.56
9	0.54	0.56
367	0.54	0.56
368	0.54	0.56
140	0.51	0.56
288	0.5	0.56
337	0.5	0.56
480	0.43	0.56
61	0.55	0.55
220	0.55	0.55
377	0.55	0.55
488	0.55	0.55
420	0.54	0.55
427	0.54	0.55
70	0.49	0.55
97	0.46	0.55
209	0.54	0.54
277	0.54	0.54
335	0.53	0.54
392	0.53	0.54
238	0.52	0.54
339	0.51	0.54
257	0.47	0.54
71	0.43	0.54
428	0.25	0.54

T1055

NMR
6zyc

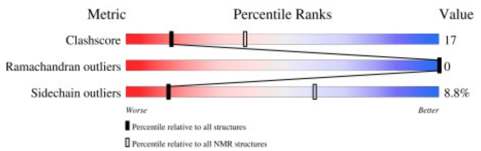

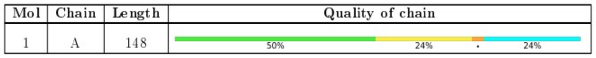

T1027

NMR
7d2o

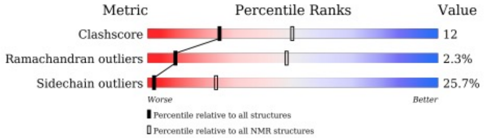

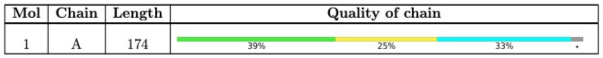

T1029

Original
NMR
6uf2

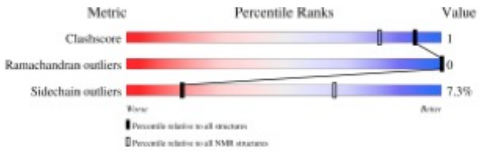

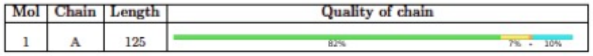

AF2

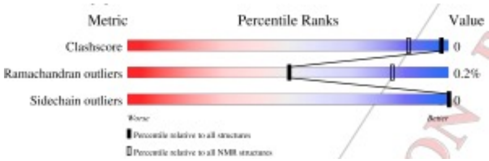

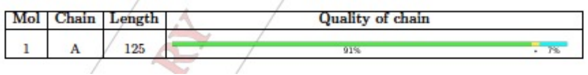

Revised
NMR
7n82

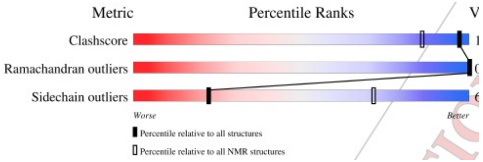

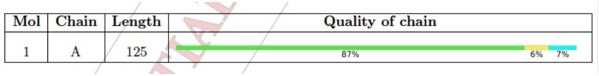

Fig. S1. wwPDB NMR structure validation summary plots

Fig. S1
